# Chemical bonding origin of the unexpected isotropic physical properties in thermoelectric Mg_3_Sb_2_ and related materials

**DOI:** 10.1038/s41467-018-06980-x

**Published:** 2018-11-09

**Authors:** Jiawei Zhang, Lirong Song, Mattia Sist, Kasper Tolborg, Bo Brummerstedt Iversen

**Affiliations:** Department of Chemistry and iNANO, Center for Materials Crystallography, Aarhus University, DK-8000 Aarhus, Denmark

## Abstract

The Mg_3_Sb_2_ structure is currently being intensely scrutinized due to its outstanding thermoelectric properties. Usually, it is described as a layered Zintl phase with a clear distinction between covalent [Mg_2_Sb_2_]^2−^ layers and ionic Mg^2+^ layers. Based on the quantitative chemical bonding analysis, we unravel instead that Mg_3_Sb_2_ exhibits a nearly isotropic three-dimensional bonding network with the interlayer and intralayer bonds being mostly ionic and surprisingly similar, which results in the nearly isotropic structural and thermal properties. The isotropic three-dimensional bonding network is found to be broadly applicable to many Mg-containing compounds with the CaAl_2_Si_2_-type structure. Intriguingly, a parameter based on the electron density can be used as an indicator measuring the anisotropy of lattice thermal conductivity in Mg_3_Sb_2_-related structures. This work extends our understanding of structure and properties based on chemical bonding analysis, and it will guide the search for and design of materials with tailored anisotropic properties.

## Abstract (web summary)

The outstanding thermoelectric Mg_3_Sb_2_ is currently being intensely scrutinized. Here, the authors reveal a nearly isotropic three-dimensional chemical bonding network as the origin of isotropic physical properties in Mg_3_Sb_2_, indicating the breakdown of the simple layered Zintl phase view.

## Introduction

Chemical bonding, as the language of chemists, paves an intuitive shortcut for understanding the structure and properties of materials^1^. One notable example is two-dimensional (2D) layered transition metal dichalcogenides, where the main feature is the weak interlayer van der Waals interaction. Due to the weak van der Waals interaction, properties such as lattice thermal conductivity generally show strong anisotropy^2,3^. There are significant studies on quantification of weak van der Waals bonding in transition metal dichalcogenides^4,5^, but the chemical bonding, especially the interlayer interaction, of many other presumed layered materials remains largely unknown.

In recent years, AB_2_X_2_ compounds crystalizing in the presumed layered CaAl_2_Si_2_ structure have attracted considerable research interest because of their promising magnetic and thermoelectric properties^6–12^. In particular, several compounds including n-type chalcogen-doped Mg_3_Sb_2_-based materials^13–17^, and p-type YbCd_1.5_Zn_0.5_Sb_2_^11^, EuZn_1__.__75_Cd_0__.__25_Sb_2_^12^, and Eu_0__.__2_Yb_0__.__2_Ca_0__.__6_Mg_2_Bi_2_^18^ were discovered to show excellent thermoelectric figures of merit larger than unity. This type of structure covers an exceptionally rich variety of compounds, where A is an alkaline-earth or a divalent rare-earth element, B is a transition metal or a main group element, and X usually belongs to group 14 and 15^19^. In general, AB_2_X_2_ with the CaAl_2_Si_2_-type structure including Mg_3_Sb_2_ is understood as a layered Zintl phase by assuming that the covalent B_2_X_2_ anionic layer receives the electrons donated by the ionic A cationic layer^10,19–21^. Zintl phases are charge-balanced compounds consisting of both covalently and ionically bonded atoms, where the ionic cations are considered as electron donors, donating electrons to the covalently-bonded anionic substructures. The covalent bonding in the anionic substructures ensures high carrier mobility, while the ionic cations allow the carrier density manipulation via doping without affecting the covalently bound network.^10,20^ This Zintl formalism has been very successful in explaining the electronic transport manipulation for promising thermoelectric materials^10,20^. The AB_2_X_2_ compounds with the CaAl_2_Si_2_-type structure are expected to show anisotropic properties due to the commonly accepted notion that the interlayer A–X interaction is much weaker than the intralayer covalent bonding in the B_2_X_2_ layer^19,21^. The structural formation and its correlation with the electronic structure in CaAl_2_Si_2_ were studied in detail by different theoretical models based on [Al_2_Si_2_]^2−^ networks with or without the effect of Ca cations^19,22–24^. In addition, electrical transport properties of CaAl_2_Si_2_-type compounds were rationalized by band structure engineering via an atomic orbital scheme^25^. Despite intensive theoretical studies on how crystal orbitals affect the electronic structure and electrical transport, there is very little knowledge on the quantitative description of chemical bonding, especially the interlayer interaction and how the chemical bonding affects thermal properties in CaAl_2_Si_2_-type compounds.

Here we report a quantitative analysis of chemical bonding in an archetypical compound Mg_3_Sb_2_ based on Bader’s quantum theory of atoms in molecules^26^, and compare it with the structurally related CaZn_2_Sb_2_ and layered van der Waals solid SnS_2_. It is found that Mg_3_Sb_2_ possesses a nearly isotropic three-dimensional (3D) chemical bonding network with the interlayer bond being mostly ionic with partial covalent nature, and comparable to the intralayer interactions. Such a unique bonding feature in Mg_3_Sb_2_ not only challenges the well-known Zintl formalism and the description as a layered structure, but also results in nearly isotropic thermal expansion coefficients, lattice compression, atomic displacement parameters, and lattice thermal conductivity. Importantly, we show how a simplified parameter based on the electron density can be used as an indicator for the anisotropy of the lattice thermal conductivity. Furthermore, the nearly isotropic 3D chemical bonding network is found to be widely applicable to many other Mg-containing compounds with the CaAl_2_Si_2_-type structure.

## Results

### Crystal structure and static deformation electron density

**Fig. 1:**
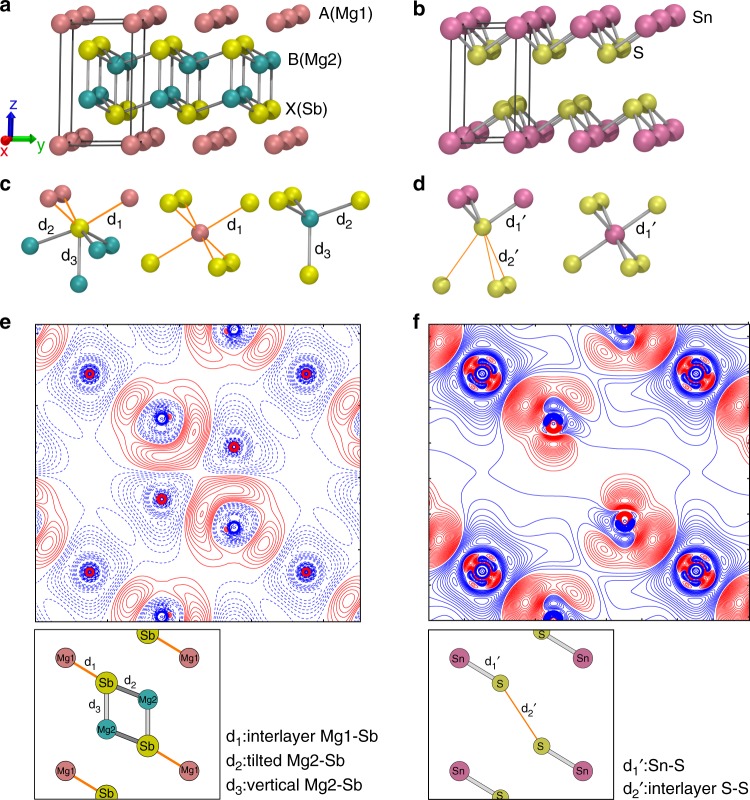
Crystal structure and static deformation electron density. **a**, **b** Crystal structure of (**a**) AB_2_X_2_ (Mg_3_Sb_2_) and (**b**) SnS_2_. Mg1 and Mg2 represent Mg atoms in the Mg monolayer and [Mg_2_Sb_2_]^2−^ layer, respectively. **c**, **d** Coordination polyhedrons of the nonequivalent atoms in (**c)** AB_2_X_2_ (Mg_3_Sb_2_) and (**d**) SnS_2_. **e**, **f** Static deformation electron density map on (110) plane containing both interlayer and intralayer interactions of (**e**) Mg_3_Sb_2_ and (**f**) SnS_2_. The contour interval is 0.006 *e* Å^−3^. Positive (negative) contours are plotted with red full (blue dotted) lines. The inset shows the corresponding (110) plane

AB_2_X_2_ with the CaAl_2_Si_2_-type structure can be described by tightly bound B_2_X_2_ anionic layers sandwiched by two-dimensional layers of A cations (Fig. 1a). Besides the interlayer A–X bond (*d*_1_), two types of bonds exist in the B_2_X_2_ slabs, i.e., the tilted and vertical B–X bond, where the vertical bond (*d*_3_) is often longer than the tilted bond (*d*_2_)^19^. Three nonequivalent atoms have completely different coordination environments: A is connected to six X atoms with six equal bonds, B is tetrahedrally coordinated by X atoms with three tilted B–X bonds and one vertical B–X bond, and X is coordinated by three A atoms and four B atoms with seven adjacent bonds including three interlayer A–X bonds, three tilted B–X bonds, and one vertical B–X bond (Fig. 1c).

Mg_3_Sb_2_ (Space group: *P*$\overline 3$*m*1, *a* = 4.56187(3) and *c* = 7.22944(6) Å at 299 K) can be considered as a special case of the CaAl_2_Si_2_ (AB_2_X_2_) structure in which A and B are Mg1 and Mg2, respectively. To have a better understanding of the interlayer interaction, a layered metal dichalcogenide SnS_2_ with the trigonal CdI_2_-type structure (Space group: *P*$\overline 3$*m*1, *a* = 3.6456(4) and *c* = 5.8934(11) Å at 300 K)^27^ was chosen for comparison since it shares many structural similarities with Mg_3_Sb_2_. Without considering the difference in lattice parameters, Mg_3_Sb_2_ can be viewed as intercalating two monolayers of Mg ions into the van der Waals gap of SnS_2_ and replacing Sn and S, respectively by Mg and Sb (Fig. 1b). Unlike the Sb atom surrounded by seven Mg atoms in Mg_3_Sb_2_, the S atom in SnS_2_ is coordinated by three Sn atoms and three S atoms with six adjacent bonds including three intralayer Sn-S bonds ($d_1\prime$) and three interlayer S–S bonds (*d*_2_′) (see Fig. 1d).

Static deformation electron density maps of the (110) planes in Mg_3_Sb_2_ and SnS_2_ are shown in Fig. 1e, f. The (110) plane is chosen because all nonequivalent bonds are included in this plane. Interestingly, in addition to the expected charge accumulations along intralayer Mg2–Sb bonds, a clear charge accumulation is observed along the interlayer Mg1-Sb bond in Mg_3_Sb_2_. The Sb atom possesses seven valence shell charge concentrations (VSCC) towards Mg atoms, including three VSCC towards Mg1 atoms and four towards Mg2 atoms (Fig. 1e, c). Comparing with the static deformation density map of SnS_2_, the charge accumulation along the interlayer Mg1–Sb is not surprising since the interlayer Mg1–Sb (*d*_1_) bond in Mg_3_Sb_2_ corresponds to the intralayer Sn–S bond (*d*_1_′) in SnS_2_. In spite of similar features of density deformation profiles between Mg1–Sb and Sn–S, much larger charge accumulation and density deformation can be seen along the Sn–S bond in SnS_2_ (Fig. 1f). This implies that the interlayer Mg1–Sb can be viewed as a weakened form of the intralayer Sn–S bond with longer bond length and less covalent nature.

### Topological analysis of electron density

Topological analysis of the theoretical full electron density was conducted based on Bader’s quantum theory of atoms in molecules^26^ (Supplementary Note 1 and Supplementary Figs. 1 and 2). Topological properties at the bond critical points (BCPs) are provided in Table 1. In Mg_3_Sb_2_, BCPs are found close to the Mg atoms along the vertical and tilted Mg2–Sb bonds and along Mg1–Sb bond (see Supplementary Fig. 1). If only based on the sign of the Laplacian ∇^2^*ρ*(**r**_b_) at the BCPs, the interlayer Mg1–Sb bond together with the two intralayer Mg2–Sb bonds can be described as closed-shell interactions or ionic bonds^28^ (see also Supplementary Fig. 3). However, the nature of chemical bonds cannot simply be judged by the sign of ∇^2^*ρ*(**r**_b_). A more accurate analysis is using the Laplacian profile in comparison with that of the Independent Atom Model (IAM). As can be seen in Supplementary Fig. 4, for all three types of bonds in Mg_3_Sb_2_ the Laplacian values along the bond path are less positive than those of IAM. This indicates the partial covalent nature in these bonds. Considering the degree of differences in Laplacian profiles, the proportion of partial covalency is slightly increasing from the interlayer Mg1-Sb to the vertical Mg2–Sb to the tilted Mg2–Sb.

**Table 1:** Topological properties of the bond critical points (r_b_)

Bond	*d* (Å)	*ρ*(r_b_) (*e* Å^−3^)	∇^2^*ρ*(r_b_) (*e* Å^−5^)	*G* (a.u.)	*V* (a.u.)	*H* (a.u.)	|*V*|/*G*	*G*/*ρ* (a.u.)
Mg_3_Sb_2_								
Interlayer Mg1–Sb	3.120	0.110	0.649	0.0075	−0.0082	−0.0008	1.101	0.460
Tilted Mg2–Sb	2.849	0.170	1.267	0.0150	−0.0168	−0.0018	1.122	0.595
Vertical Mg2–Sb	2.959	0.154	0.997	0.0122	−0.0140	−0.0018	1.150	0.533
CaZn_2_Sb_2_								
Interlayer Ca–Sb	3.230	0.122	0.831	0.0093	−0.0100	−0.0007	1.074	0.516
Tilted Zn–Sb	2.719	0.313	0.734	0.0223	−0.0370	−0.0147	1.659	0.480
Vertical Zn–Sb	2.820	0.259	0.738	0.0177	−0.0277	−0.0100	1.567	0.460
SnS_2_								
Interlayer S–S	3.609	0.051	0.485	0.0042	−0.0034	0.0008	0.801	0.553
Sn–S	2.592	0.424	1.512	0.0390	−0.0624	−0.0233	1.598	0.620

Note: *d* is the bond length. *ρ*(**r**_b_) and ∇^2^*ρ*(**r**_b_) are the charge density and its Laplacian at the BCP, respectively. *G* and *V* denote the kinetic and potential energy density at the BCP, respectively. *H* is the total energy density (*H* = *G* + *V*). *G*, *V*, *H*, and *G*/*ρ* are in a.u.

According to the well-established classification scheme^28^, the interlayer Mg1-Sb and intralayer Mg2-Sb bonds can be described as polar bonds according to the BCPs properties with the small density *ρ*(**r**_b_), positive ∇^2^*ρ*(**r**_b_), |*V*|/*G* being slightly larger than unity, negative total energy density *H*, and *G*/*ρ* < 1 (see Table 1). Changing from the tilted Mg2–Sb to the vertical Mg2–Sb to the Mg1–Sb bond, the density value undergoes a negligible decrease, which indicates a minor decrease in covalency and interaction strength. In contrast, a remarkable difference is observed between the interlayer S–S and intralayer Sn–S bonds in SnS_2_ (see Table 1). The interlayer S–S interaction can be described as a weak van der Waals bond as judged by the very small electron density *ρ*(**r**_b_), positive ∇^2^*ρ*(**r**_b_), |*V*|/*G* < 1, and positive *H* at the BCP, whereas the intralayer Sn–S interaction can be treated as a polar covalent bond based on the positive ∇^2^*ρ*(**r**_b_), 1 < |*V*|/*G* < 2, negative *H*, and *G*/*ρ* *<* 1. The electron density at the BCP of the interlayer S–S bond is approximately 8 times smaller than that of the Sn–S bond, indicating the much weaker strength of the interlayer interaction compared with that of the intralayer interaction. Upon comparison of topological properties at the BCPs (see Table 1), the three similar bonds in Mg_3_Sb_2_ are clearly more polar and weaker than the Sn–S bond in SnS_2_, but they are stronger than the weak interlayer S–S interaction.

The topological properties at the BCPs of another archetypical CaAl_2_Si_2_-type compound, CaZn_2_Sb_2_, are analyzed and compared with those of Mg_3_Sb_2_ (see Table 1). Clearly, the interlayer interactions are quantitatively similar, but the intralayer interactions in B_2_X_2_ slabs show significant differences. The intralayer Zn–Sb bonds in CaZn_2_Sb_2_, described as polar covalent bonds, show much larger values of *ρ*(**r**_b_), |*V*|/*G*, and –*H*, which denotes that the Zn–Sb bonds are much more covalent and stronger than the interlayer Ca-Sb bond and the intralyer Mg2–Sb bonds in Mg_3_Sb_2_. This is consistent with the smaller electronegativity difference and the shorter bond distance between Zn and Sb. Despite the stronger Zn–Sb interaction, the difference between the interlayer and intralayer interactions in CaZn_2_Sb_2_ is moderate if compared to that of SnS_2_.

The above deduction is further strengthened by the values of the Bader atomic properties shown in Table 2. The atomic charges of Zn and Sb in CaZn_2_Sb_2_ being far from the nominal oxidation states suggest a high degree of covalency in the [Zn_2_Sb_2_]^2−^ slabs, whereas the large charge transfer of the Ca atom indicates largely ionic features in the Ca^2+^ layers. In contrast, nearly complete charge transfers are observed for all atoms in Mg_3_Sb_2_ with Mg1 and Mg2 showing nearly the same atomic charges, which indicates that the interlayer and intralayer bonds are largely ionic and comparable. Based on the above results, the description of a layered Zintl phase holds true for CaZn_2_Sb_2_ since it meets the assumption that the ionic A^2+^ layer donates electrons to the covalent [B_2_X_2_]^2−^ layer; however, this description is not applicable to Mg_3_Sb_2_ since the intralayer bonds in the [Mg_2_Sb_2_]^2−^ slabs are not really covalent. Therefore, since the chemical bonds in Mg_3_Sb_2_ are largely ionic without a clear distinction of ionic and covalent parts we approach the limit of the application of the well-recognized Zintl formalism in CaAl_2_Si_2_-type compounds.

**Table 2:** Atomic properties

Atoms	*Q* (e)	*V* (Å^3^)
Mg_3_Sb_2_		
Mg1	1.51	8.71
Mg2	1.47	8.50
Sb	−2.23	53.69
CaZn_2_Sb_2_		
Ca	1.37	15.71
Zn	0.31	18.74
Sb	−0.99	38.85
SnS_2_		
Sn	1.53	15.40
S	−0.76	26.81

*Q* and *V* represent the atomic charge and the atomic basin volume, respectively

### Non-covalent interaction analysis

The non-covalent interaction (NCI) index, based on the electron density, *ρ*, and its derivatives, is a powerful tool to reveal weak interlayer interactions^29^. NCI analysis is based on the reduced density gradient (RDG) as a function of sign(*λ*_2_)*ρ* (see methods), where sign(*λ*_2_) is the sign of the second eigenvalue of the electron density Hessian matrix^29,30^. Negative values of sign(*λ*_2_)*ρ* indicate attractive interactions, whereas positive values suggest repulsive interaction. Spikes induced by the significant change in RDG approaching zero at critical points within low density regions correspond to weak interactions. The density value of the spike with low RDG relates to the strength of the corresponding interaction.

**Fig. 2:**
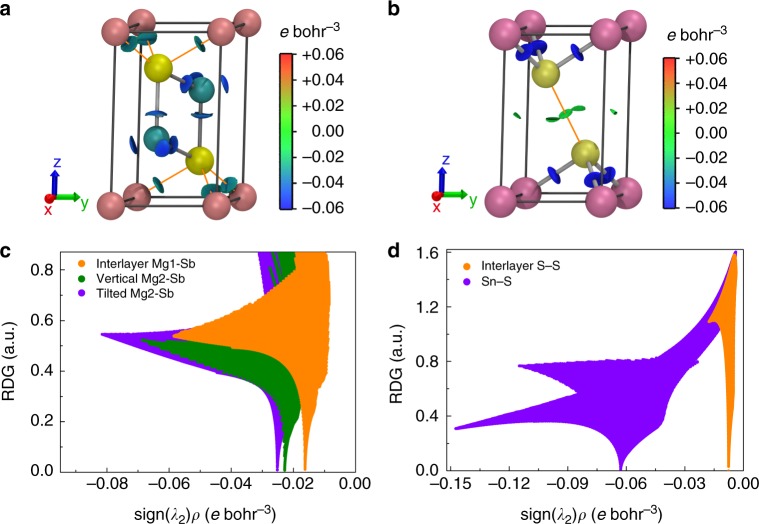
Non-covalent interaction analysis. **a**, **b** 3D non-covalent interaction plot for (**a**) Mg_3_Sb_2_ and (**b**) SnS_2_. The RDG isosurface corresponds to RDG = 0.24 a.u., which is colored on a BGR scale of −0.06 < sign(*λ*_2_)*ρ* < 0.06 *e* bohr^−3^. **c**, **d** RDG as a function of sign(*λ*_2_)*ρ* for the interlayer and intralayer interactions in (**c**) Mg_3_Sb_2_ and (**d**) SnS_2_

3D RDG isosurfaces with blue-green-red (BGR) color scales representing sign(*λ*_2_)*ρ* values are given in Fig. 2a, b. Dark green RDG isosurfaces indicate that the interlayer Mg1–Sb in Mg_3_Sb_2_ is an attractive interaction, stronger than the weak interlayer S–S interaction in SnS_2_ with RDG isosurfaces colored in green. In order to quantitatively understand the interlayer interactions, the dependence of RDG on sign(*λ*_2_)*ρ* is calculated and shown in Fig. 2c, d. As expected, distinct differences can be seen between the weak interlayer S–S and intralayer Sn–S interactions in SnS_2_. Compared with the intralayer Sn–S interaction, the interlayer S–S interaction shows a low RDG peak with a much smaller sign(*λ*_2_)*ρ* value approaching zero, a clear indication of weak van der Waals interaction (Fig. 2d). In contrast, the RDG distribution of the interlayer Mg1–Sb interaction in Mg_3_Sb_2_ is very similar to those of the intralayer Mg2-Sb interactions (Fig. 2c). The density value of the low RDG peak for the interlayer Mg1–Sb interaction is just slightly lower than those of the vertical and tilted Mg2–Sb intralayer interactions. This further confirms that the interlayer and intralayer interactions in Mg_3_Sb_2_ are comparable; that is, the tilted Mg2–Sb bond is slightly stronger than the vertical Mg2–Sb bond, while the vertical Mg2–Sb bond is slightly stronger than the interlayer Mg1-Sb interaction.

The comprehensive chemical bonding analysis paves the way to understand structure and properties. The largely ionic feature with partial covalency (high polarity) of chemical bonds in Mg_3_Sb_2_ explains the intrinsically poor carrier density and mobility, as well as the reasonably low lattice thermal conductivity^31^. Furthermore, the comparable interlayer and intralayer interactions unveil the three-dimensional chemical bonding network in Mg_3_Sb_2_, ruling out the description as a typical layered structure. Importantly, the 3D bonding network is nearly isotropic in Mg_3_Sb_2_ upon the quantitative comparisons of topological properties of different bonds. Such a feature is decisive for many unique properties including structural parameters and thermal properties.

### Lattice thermal expansion and pressure compression

**Fig. 3:**
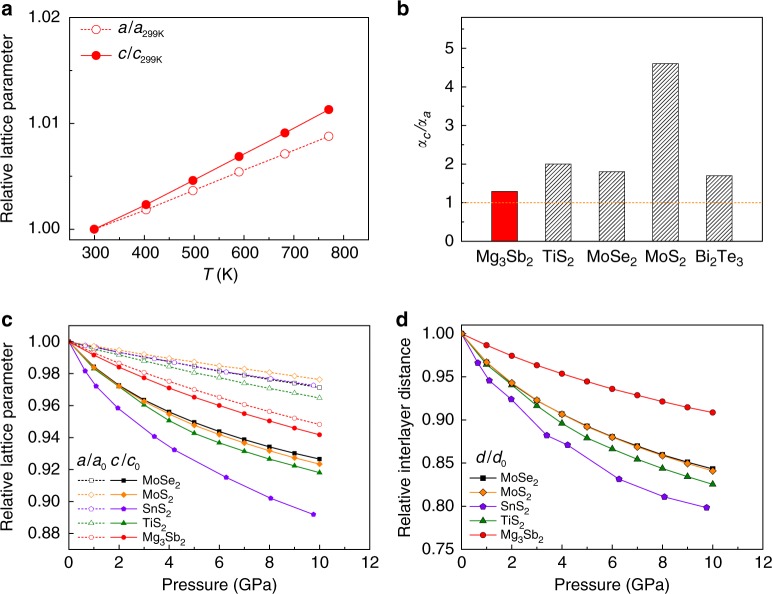
Lattice thermal expansion and pressure compression. **a** Relative lattice constants *a*/*a*_299K_ and *c*/*c*_299K_ of Mg_3_Sb_2_ as a function of temperature. The data are obtained from Rietveld refinement of the multi-temperature synchrotron PXRD. **b** Anisotropic ratio of the linear thermal expansion coefficient *α*_*c*_/*α*_*a*_ in Mg_3_Sb_2_ compared to those of several typical layered materials, that is, TiS_2_^32^, MoSe_2_^33^, MoS_2_^33^, and Bi_2_Te_3_^34^. **c**, **d** The calculated pressure dependence of (**c**) the relative lattice parameters and (**d**) the relative interlayer distance of Mg_3_Sb_2_ in comparison with those of several typical layered metal dichalcogenides. The data of SnS_2_ are adapted from the reported experimental work^27^

Chemical bonding analysis is crucial for understanding the lattice response under physical conditions such as temperature and pressure. Figure 3a shows the temperature dependence of the experimental lattice parameters of Mg_3_Sb_2_ (see also Supplementary Fig. 5 and Supplementary Table 1). As illustrated in the figure, the lattice parameters all display linear increasing trends as the temperature increases and the thermal expansion along the *c* axis is slightly larger than that along the *a* axis. The linear thermal expansion coefficients at room temperature along the *a* and *c* directions in Mg_3_Sb_2_ are, respectively, 1.88 × 10^−5^ and 2.42 × 10^−5^ K^−1^, which leads to a nearly isotropic *α*_*c*_*/α*_*a*_ of 1.29, much smaller than those of typical layered materials TiS_2_^32^, MoS_2_^33^, MoSe_2_^33^, and Bi_2_Te_3_^34^ (see Fig. 3b and Supplementary Table 2). Furthermore, similar results are observed in the pressure-induced lattice compression. The relative lattice parameters and interlayer distance as a function of a series of hydrostatic pressures are simulated using density functional theory (DFT) calculations and given in Fig. 3c, d. Under the same pressure, the decrease of the relative lattice parameter *c*/*c*_0_ in Mg_3_Sb_2_ is just slightly larger than that of *a*/*a*_0_, whereas the lattice parameter *c* is much more compressible than *a* in all layered van der Waals solids SnS_2_^27^, TiS_2_, MoS_2_, and MoSe_2_.

It is clear that both the lattice expansion with temperature and the lattice compression under pressure exhibit nearly isotropic features in Mg_3_Sb_2_, which can be essentially understood by the 3D chemical bonding network in this material. In both cases, the lattice parameter *c* in Mg_3_Sb_2_ shows slightly larger thermal expansion or pressure compression than that of *a*, which can be attributed to the slightly weaker interlayer bond compared with the intralayer bonds. Moreover, the interlayer distance of Mg_3_Sb_2_ is less compressible than those of van der Waals solids, confirming the interlayer interaction being stronger than the van der Waals force.

### Atomic displacement parameters and potential energy curves

**Fig. 4:**
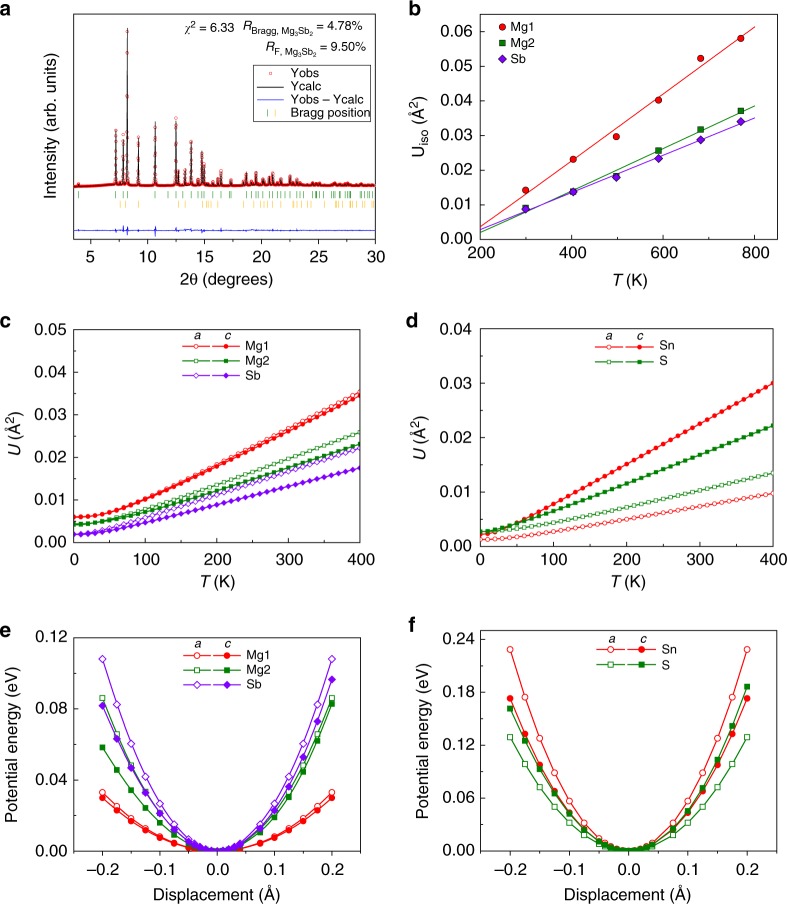
Atomic displacement parameters and potential energy curves. **a** Calculated (Rietveld method) and observed synchrotron PXRD patterns of Mg_3_Sb_2_ at 770 K upon cooling. Red open circles and black line are observed and calculated data, respectively. The blue line represents the difference between the observed and calculated patterns. The green and orange vertical bars correspond to the Bragg positions of the main phase Mg_3_Sb_2_ and the secondary phase Sb, respectively. **b** Temperature dependence of the experimental isotropic atomic displacement parameters of Mg_3_Sb_2_. **c**, **d** Temperature dependence of the theoretical atomic displacement parameters along different axial directions of (**c**) Mg_3_Sb_2_ and (**d**) SnS_2_. **e**, **f** The potential energy curves for the nonequivalent atoms of (**e**) Mg_3_Sb_2_ and (**f**) SnS_2_ as a function of the displacements from the equilibrium positions

To gain insight on how chemical bonding affects the thermal motion of the atoms, the isotropic atomic displacement parameters *U*_iso_ of Mg_3_Sb_2_ were obtained from Rietveld refinement of multi-temperature synchrotron powder X-ray diffraction (PXRD) data (Fig. 4a). Refinement details are provided in Supplementary Table 1 and Supplementary Note 2. As illustrated in Fig. 4b, the Mg1 atoms exhibit larger thermal displacements than those of the Mg2 and Sb atoms. This experimental trend is well reproduced by the theoretical result based on the harmonic approximation shown in Fig. 4c. The atomic thermal motions are closely related to the potential energy surfaces (see Fig. 4e). By displacing the atoms from their equilibrium positions, we found that the Mg1 atom shows a relatively flat potential well and it is thereby loosely bonded, consistent with its relatively large thermal vibration. In fact, the larger thermal displacement and flatter potential originate from the weaker adjacent bonds (i.e., the interlayer Mg1-Sb bonds) of the Mg1 atom. In addition, a slightly larger *U*_iso_ of Mg2 than that of Sb in the [Mg_2_Sb_2_]^2−^ slabs was observed at elevated temperatures, which can be rationalized by the difference in atomic masses.

When considering the atomic displacement parameters and potential wells along the axial directions, both the Sn and S atoms in layered SnS_2_ display highly anisotropic characteristics with the vibration along the *c* direction being significantly larger than along the *a* direction (see Fig. 4d, f). This is due to the weak van der Waals interaction along the *c* axis. However, the atomic displacement parameters and potential wells of all atoms in Mg_3_Sb_2_ are relatively isotropic along different axial directions (Fig. 4c). The Mg1 atom manifests perfect isotropic features due to its equally adjacent Mg1–Sb bonds, while the Mg2 and Sb atoms show less isotropic features in atomic displacement parameters because of their comparable but nonequivalent adjacent bonds. The above result is another validation for the notion of comparable interlayer and intralayer bonding interactions in Mg_3_Sb_2_. Furthermore, it is found that the potential wells of all atoms except Mg2 and Sb along the *c* direction are ideally harmonic, which can be reasonably understood by the atoms being surrounded by symmetric electron density along the axial directions (see Supplementary Figs. 6a and 7). However, slightly anharmonic features can be seen in potential wells of the Mg2 and Sb atoms along the *c* direction (see Supplementary Fig. 6b), which is induced by the different strengths of the three nonequivalent bonds in Mg_3_Sb_2_.

### Lattice thermal conductivity

**Fig. 5:**
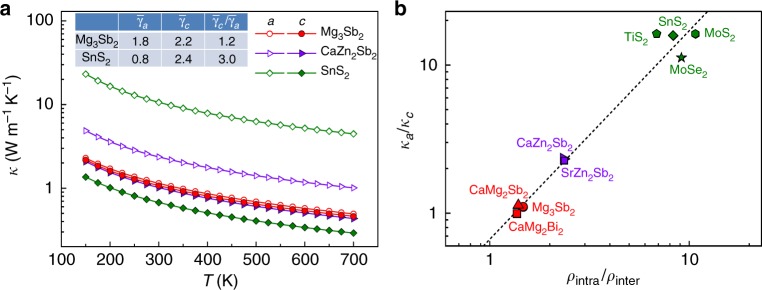
Lattice thermal conductivity. **a** Calculated lattice thermal conductivity of Mg_3_Sb_2_, CaZn_2_Sb_2_, and SnS_2_ along *a* and *c* directions as a function of temperature. The inset table displays the average Grüneisen parameters at room temperature along *a* and *c* directions. **b** Anisotropy of lattice thermal conductivity *κ*_*a*_/*κ*_*c*_ at 300 K as a function of the intralayer-to-interlayer bond-strength ratio *ρ*_intra_/*ρ*_inter_. *ρ*_intra_ and *ρ*_inter_ represent the electron density values at BCPs of the intralayer and interlayer bonds, respectively. The theoretical lattice thermal conductivity data of MoSe_2_ and MoS_2_ are adapted from ref. ^3^. The dashed line is a guide to the eyes

It is well known that weak chemical bonding usually accompanied by strong lattice anharmonicity leads to low lattice thermal conductivity^35,36^. To probe the effect of chemical bonding on thermal transport, the lattice thermal conductivity was simulated by DFT calculations. Indeed, weak van der Waals interaction between the layers in SnS_2_ leads to considerably lower lattice thermal conductivity along the *c* axis in comparison with that along the *a* axis (see Fig. 5a). High anisotropic ratio *κ*_*a*_/*κ*_*c*_ of lattice thermal conductivity is commonly observed at 300 K in typical layered materials, such as 15.8 in SnS_2_, 16.2 in TiS_2_, 16.2 in MoS_2_^3^, and 11.2 in MoSe_2_^3^ (Fig. 5b). Moreover, the anisotropic lattice thermal conductivity (*κ*_*a*_/*κ*_*c*_ ≈ 2.3) is found in layered Zintl phases AZn_2_Sb_2_ (A = Ca and Sr). In contrast, unlike the noticeable anisotropy in van der Waals solids and AZn_2_Sb_2_, the lattice thermal conductivity in Mg_3_Sb_2_ is nearly isotropic with *κ* along the *c* axis being negligibly lower than that along the *a* axis (*κ*_*a*_/*κ*_*c*_ ≈ 1.1 at 300 K, see Fig. 5).

In order to elucidate the origin of the isotropic lattice thermal conductivity in Mg_3_Sb_2_, phonon dispersion, group velocity, and Grüneisen parameter were calculated. The Grüneisen parameter, defined as the response of phonon frequencies to volume change, represents the strength of the lattice anharmonicity. The average Grüneisen parameters along the *a* and *c* axes in Mg_3_Sb_2_ are 1.8 and 2.2, respectively, which gives an anisotropic ratio of ~1.2, much smaller than that of ~3.0 in layered SnS_2_ (see the inset table of Fig. 5a). The slightly higher Grüneisen parameter, as well as the aforementioned weak anharmonic potential wells along the *c* axis in Mg_3_Sb_2_ induced by the slightly weaker interlayer Mg1–Sb bond, explains the smaller lattice thermal conductivity along this direction. In addition to the Grüneisen parameter, the phonon dispersion and group velocity also show nearly isotropic features in Mg_3_Sb_2_, whereas those in layered SnS_2_ are considerably anisotropic (see Supplementary Fig. 8 and Supplementary Table 3).

The origin of anisotropy in thermal properties can be traced to the chemical bonding. We can define a simplified parameter, the intralayer-to-interlayer bond-strength ratio *ρ*_intra_/*ρ*_inter_, which measures the degree of anisotropy of the chemical bonding network in a presumed layered structure. *ρ*_intra_ and *ρ*_inter_ denote the electron density values at the BCPs of the intralayer and interlayer bonds, respectively. For the AB_2_X_2_ compounds with two nonequivalent intralayer bonds, *ρ*_intra_ is calculated by averaging the electron density values at BCPs of the two intralayer bonds. As can be seen in Fig. 5b, a nearly linear correlation between the anisotropic ratio *κ*_*a*_/*κ*_*c*_ of lattice thermal conductivity and *ρ*_intra_/*ρ*_inter_ is revealed. This suggests that *ρ*_intra_/*ρ*_inter_ can be adopted as an indicator for the anisotropy of lattice thermal conductivity. *ρ*_intra_/*ρ*_inter_≈1 in Mg_3_Sb_2_ indicates a nearly isotropic 3D chemical bonding network, which results in the nearly isotropic feature in phonon dispersion, group velocity, Grüneisen parameter, and eventually in lattice thermal conductivity.

It should be noted that the nearly isotropic 3D bonding network is not limited to Mg_3_Sb_2_. The topological properties of several other Mg-containing compounds including Mg_3_Bi_2_, CaMg_2_Sb_2_, CaMg_2_Bi_2_, SrMg_2_Sb_2_, and YbMg_2_Sb_2_ are shown in Supplementary Tables 4-6. All these compounds show comparable interlayer and intralayer polar bonds with *ρ*_intra_/*ρ*_inter_≈1, similar to those of Mg_3_Sb_2_. This suggests that the nearly isotropic 3D bonding network is a general feature in AMg_2_X_2_ compounds with the CaAl_2_Si_2_-type structure.

## Discussion

In summary, using quantitative analysis of chemical bonding, we have shown that the interlayer interaction in Mg_3_Sb_2_ is largely ionic with partial covalent nature, and it exhibits the same type of interaction with comparable strength as the intralayer chemical bonds. This result not only indicates that Mg_3_Sb_2_ cannot be described as a layered structure, but also challenges the widely accepted Zintl concept that assumes the [Mg_2_Sb_2_]^2−^ slabs being covalently bonded. The nearly isotropic 3D bonding network formed by the comparable chemical bonds leads to the isotropic characteristics in many properties, such as lattice thermal expansion, lattice compression under hydrostatic pressure, atomic displacement parameters, and lattice thermal conductivity. Interestingly, the intralayer-to-interlayer bond-strength ratio based on the electron density is established as a simplified descriptor for the anisotropy of lattice thermal conductivity. Moreover, it is found that the nearly isotropic 3D chemical bonding network is not limited to Mg_3_Sb_2_ but can be broadly applied to many other Mg-containing materials with the CaAl_2_Si_2_ structure. Thus, this work extends our fundamental understanding of the structure-property relationship using chemical bonding as a bridge and it will guide the rational design of materials with tailored properties.

## Methods

### Sample synthesis

Mg_3_Sb_2_ crystals were synthesized from the mixture of high-purity elements Sb pieces (99.9999%, Chempur) and Mg turnings (99.5%, Chempur). 0.8 mole excess Mg was added to compensate the evaporation loss during the synthesis. The mixture of Mg and Sb was loaded into a glassy carbon crucible with a lid. The crucible was placed in a quartz tube, which was evacuated to a pressure smaller than 10^−4^ mbar and flame-sealed. Mg_3_Sb_2_ crystals were obtained using vertical Bridgman crystal growth by heating to 973 K for 24 h and then slowly cooling down to room temperature over 160 h with the sample moving at a rate of 2 mm h^−1^.

### Synchrotron powder X-ray diffraction

Mg_3_Sb_2_ powders, obtained by crushing the crystals, were floated in ethanol in order to select very small and homogeneous particles. The obtained fine powders were packed under Ar in a 0.2 mm diameter quartz capillary. Synchrotron PXRD patterns were collected at SPring-8 BL44B2 beamline^37^ with a wavelength of 0.500197(1) Å, which was calibrated by a CeO_2_ standard. Data were collected at 299–813 K (heating) and 770–299 K (cooling) with the high temperature setup (see Supplementary Fig. 9). Synchrotron PXRD patterns were analyzed using Rietveld refinement in the FullProf program^38^. The peak profiles were described by the Thompson-Cox-Hastings pseudo-Voigt function^39^ convoluted with axial divergence asymmetry. The background was modeled using a linear interpolation between a set of manually selected background points. The detailed results of the refinements of all heating and cooling data were provided in Supplementary Tables 1, 2, and 7–9. The analysis of the data at 770–299 K upon cooling was used for discussion in the main text.

### Theoretical calculations

DFT calculations in this work were performed in the Wien2k code^40^ using a full-potential linear augmented plane-wave plus local orbitals method and with the VASP code^41^ based on the projector-augmented wave method^42^. The structural parameters including lattice parameters and atomic positions were fully relaxed until the Hellmann-Feynman force criterion of 0.001 eV Å^−1^ was reached in the VASP code. For the structural relaxations, Monkhorst-Pack *k*-point meshes of 6 × 6 × 4, 9 × 9 × 6, and 8 × 8 × 2 were adopted for the CaAl_2_Si_2_-type compounds, TiS_2_ (SnS_2_), and MoS_2_ (MoSe_2_), respectively. All calculations for Mg_3_Sb_2_, CaZn_2_Sb_2_, and other compounds with the CaAl_2_Si_2_-type structure were performed using the PBE functional^43^, whereas the calculations for layered SnS_2_, TiS_2_, MoS_2_, and MoSe_2_ were carried out using van der Waals functional optB86b-vdW^44^. Here we use optB86b-vdW functional for metal dichalcogenides since it gives structural parameters under hydrostatic pressure in good agreement with those from experiment^27^. Structural parameters of Mg_3_Sb_2_, TiS_2_, MoS_2_, and MoSe_2_ under hydrostatic pressure were calculated in this work, while the high-pressure data of SnS_2_ are from a reported experimental work^27^. An energy convergence criterion of 10^−6^ eV and a plane wave energy cutoff of 500 eV were adopted for calculations.

The electron density calculations were done with the Wien2k code on a dense 22 × 22 × 12 k mesh with the plane wave cutoff parameter *R*_MT_*K*_max_ of 10. The relaxed structural parameters were used for the calculations. The charge density inside the atomic spheres was expanded to form spherical harmonics up to *l*_max_ = 10. The muffin-tin radii (*R*_MT_) for Mg, Sb, Sn, and S were chosen as 2.3, 2.5, 2.0, and 1.8 bohr, respectively. The topological analysis of the total electron density based on Bader’s quantum theory of atoms in molecules was conducted with the Critic2 code^45,46^. Atomic charges and atomic basin volumes were calculated using the QTREE algorithm^47^ as implemented in the Critic2 code. Non-covalent interaction (NCI) plots were computed with the NCIPLOT program^29,30^. NCI analysis is based on the electron density *ρ* and its reduced density gradient (RDG). The RDG can be expressed as^29,30^

1 $${\mathrm{RDG}} = \frac{1}{{2\left( {3{\mathrm{\pi }}^2} \right)^{1/3}}}\frac{{\left| {\nabla \rho } \right|}}{{\rho ^{4/3}}}.$$

The 3D NCI plot with RDG isosurfaces in real space was visualized by VMD^48^. The isosurfaces were colored according to the value of sign(*λ*_2_)*ρ*, where a BGR color scale was adopted. Blue color represents attractive or bonding interaction, green weak van der Waals interaction, and red repulsive interaction. To have a clear view of the interlayer and intralayer interactions, the isosurfaces with sign(*λ*_2_)*ρ* > 0 are eliminated in Fig. 2a, b.

The harmonic phonon dispersion and atomic displacement parameters were computed with VASP and Phonopy^49^ using the finite displacement method^50^. The default displacement amplitude of 0.01 Å was used for Mg_3_Sb_2_ and SnS_2_. The calculations were done in 4 × 4 × 2 supercells to balance the computational cost and well-converged phonon dispersions (see Supplementary Fig. 10). The results of phonon dispersions for Mg_3_Sb_2_ and SnS_2_ are shown in Supplementary Fig. 8. ±5% of the equilibrium volume was used for the calculation of mode Grüneisen parameter. The average Grüneisen parameter was calculated using

2 $$\bar \gamma {\mathrm{ = }}\frac{{\mathop {\sum}\limits_{{\mathbf{q}},i} {\gamma \left( {{\mathbf{q}},i} \right)} \,C_V\left( {{\mathbf{q}},i} \right)}}{{\mathop {\sum}\limits_{{\mathbf{q}},i} {C_V\left( {{\mathbf{q}},i} \right)} \,}},$$

where *γ*(**q**,*i*) is the mode Grüneisen parameter for the phonon branch *i* at wave vector **q**, given as

3 $$\gamma \left( {{\mathbf{q}},i} \right){\mathrm{ = }} - \frac{V}{{\omega \left( {{\mathbf{q}},i} \right)}}\frac{{\partial \omega \left( {{\mathbf{q}},i} \right)}}{{\partial V}},$$

where *ω*(**q**,*i*) is phonon frequency, *V* is volume, and *C*_*V*_(**q**,*i*) is the mode dependent heat capacity defined as

4 $$C_V\left( {{\mathbf{q}},i} \right) = k_{\mathrm{B}}\left( {\frac{{\hbar \omega ({\mathbf{q}},i)}}{{k_{\mathrm{B}}T}}} \right)^2\frac{{e^{\hbar \omega ({\mathbf{q}},i)/k_{\mathrm{B}}T}}}{{\left( {e^{\hbar \omega ({\mathbf{q}},i)/k_{\mathrm{B}}T} - 1} \right)^2}}.$$

Another averaging method of Grüneisen parameter and the corresponding result are shown in Supplementary Note 3 and Supplementary Table 10, respectively. Moreover, group velocities along the axial directions for the acoustic branches were computed and they are shown in Supplementary Table 3. The lattice thermal conductivity was computed with the ShengBTE code based on a full iterative solution to the Boltzmann transport equation for phonons^51^. The second-order interatomic force constants were computed in the 4 × 4 × 2 supercells with a Monkhorst-Pack *k* mesh of 3 × 3 × 4. Considering the computational cost, the third-order interatomic force constants were calculated in the 4 × 4 × 2 supercells with a 3 × 3 × 4 Monkhorst-Pack *k* mesh for SnS_2_ and the 3 × 3 × 3 supercells with a 3 × 3 × 2 Monkhorst-Pack *k* mesh for Mg_3_Sb_2_, CaMg_2_Sb_2_, CaMg_2_Bi_2_, CaZn_2_Sb_2_, and SrZn_2_Sb_2_. To ensure well-converged thermal conductivity (see Supplementary Fig. 11), the interaction range up to the seventh nearest neighbors was considered for the calculations of the third-order interatomic force constants. The calculation details of lattice thermal conductivity of another metal dichalcogenide TiS_2_ are also provided in Supplementary Note 3 and Supplementary Fig. 12.

## Electronic supplementary material

Supporting Information

Peer Review File

## Data Availability

The data that support the findings of this study are available from the corresponding author upon reasonable request.
