# Supplementary material for: Chemical bonding origin of the unexpected isotropic physical properties in thermoelectric Mg3Sb2 and related materials
Source: Nat Commun. 2018 Nov 9;9:4716. doi: 10.1038/s41467-018-06980-x (PMC6226478; doi:10.1038/s41467-018-06980-x)
Supplement: Supplementary file 1 — Supporting Information [file 41467_2018_6980_MOESM1_ESM.pdf]

Supplementary Information for

Chemical bonding origin of the unexpected isotropic physical properties in thermoelectric Mg_3Sb_2 and related materials

*Jiawei Zhang, Lirong Song, Mattia Sist, Kasper Tolborg, and Bo Brummerstedt Iversen**

Center for Materials Crystallography, Department of Chemistry and iNANO, Aarhus University, DK-8000 Aarhus, Denmark

*Corresponding author: bo@chem.au.dk

Including:

Supplementary Figures 1-12

Supplementary Tables 1-10

Supplementary Notes 1-3

Supplementary References 1-13

Supplementary Figures

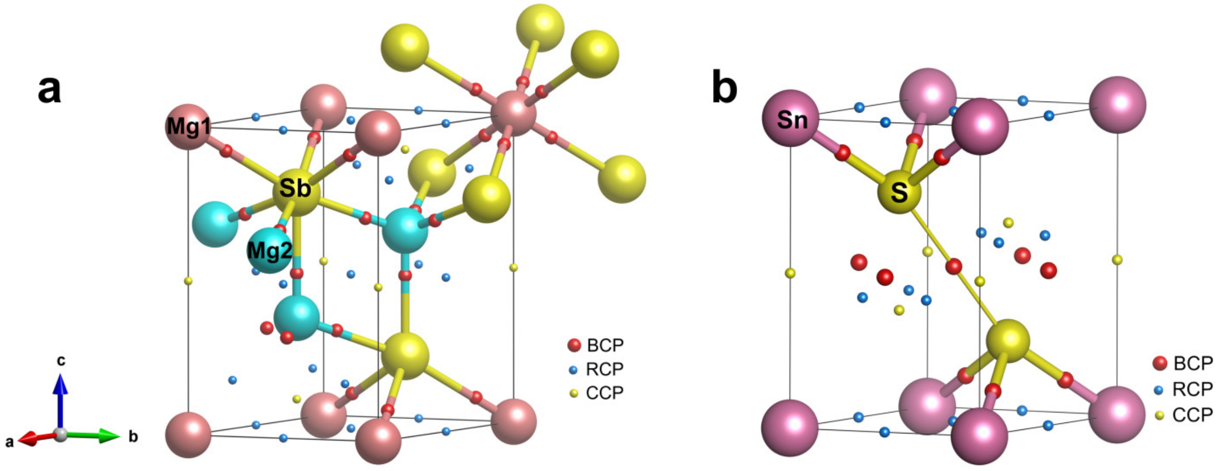

Supplementary Figure 1 | Illustration of critical points of electron density. 3D plot of critical points of electron density in (a) Mg_3Sb_2 and (b) SnS_2. BCP, RCP, and CCP denote bond critical point, ring critical point, and cage critical point, respectively.

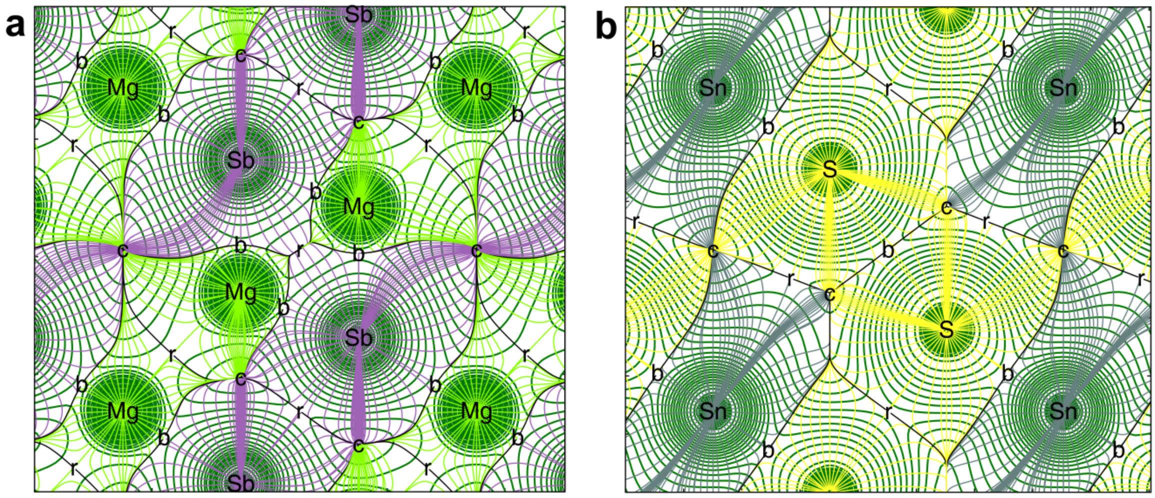

Supplementary Figure 2 | Trajectories of the gradients of electron density. Total electron density map with zero-flux surfaces and trajectories of the gradients on the (110) plane of (a) Mg_3Sb_2 and (b) SnS_2. r, b, and c denote ring critical point, bond critical point, and cage critical point, respectively. The olive and black curves represent the total electron density contours and zero-flux curves, respectively. The green, purple, yellow, and dark green lines denote the gradient paths.

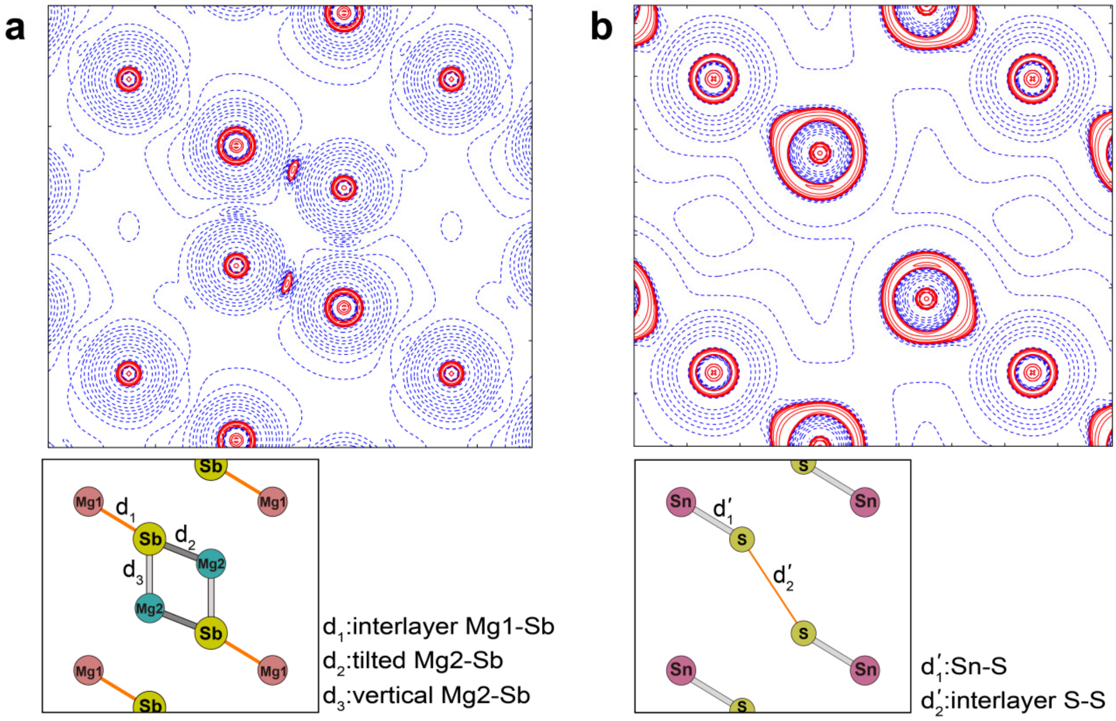

Supplementary Figure 3 | Negative Laplacian map of electron density. Negative Laplacian map on (110) plane of (a) Mg_3Sb_2 and (b) SnS_2. Contours are drawn at $\pm 2\times10^n$, $\pm 4\times10^n$ and $\pm 8\times10^n$ e/Å^5 (n = ±3, ±2, ±1, 0). Red solid and blue dotted lines represent positive and negative values, respectively. The inset shows the corresponding (110) plane.

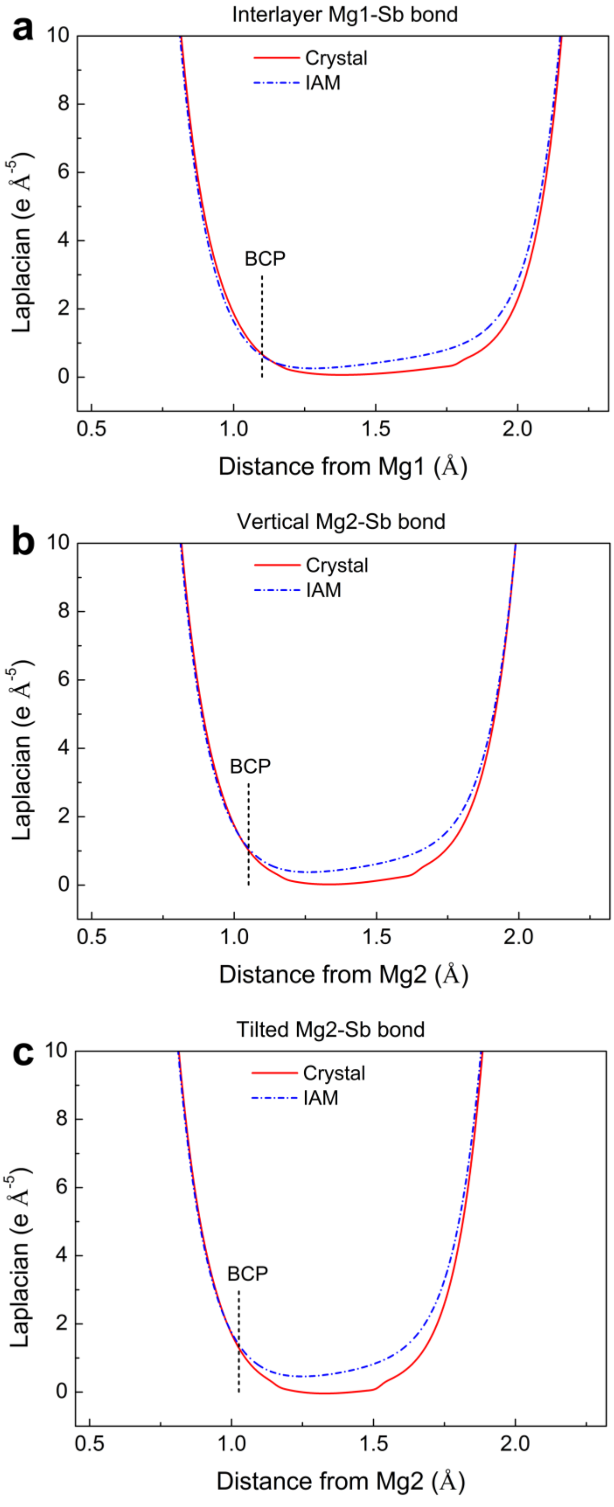

Supplementary Figure 4 | Laplacian profiles along the bond paths. Line plots of Laplacian along the bond paths of (a) Mg1-Sb, (b) vertical Mg2-Sb, and (c) tilted Mg2-Sb. The red solid line represents the result based on the full electron density of Mg_3Sb_2, whereas the dash dot line is the result based on the electron density of the Independent Atom Model (IAM). The vertical dash line marks the location of the bond critical point.

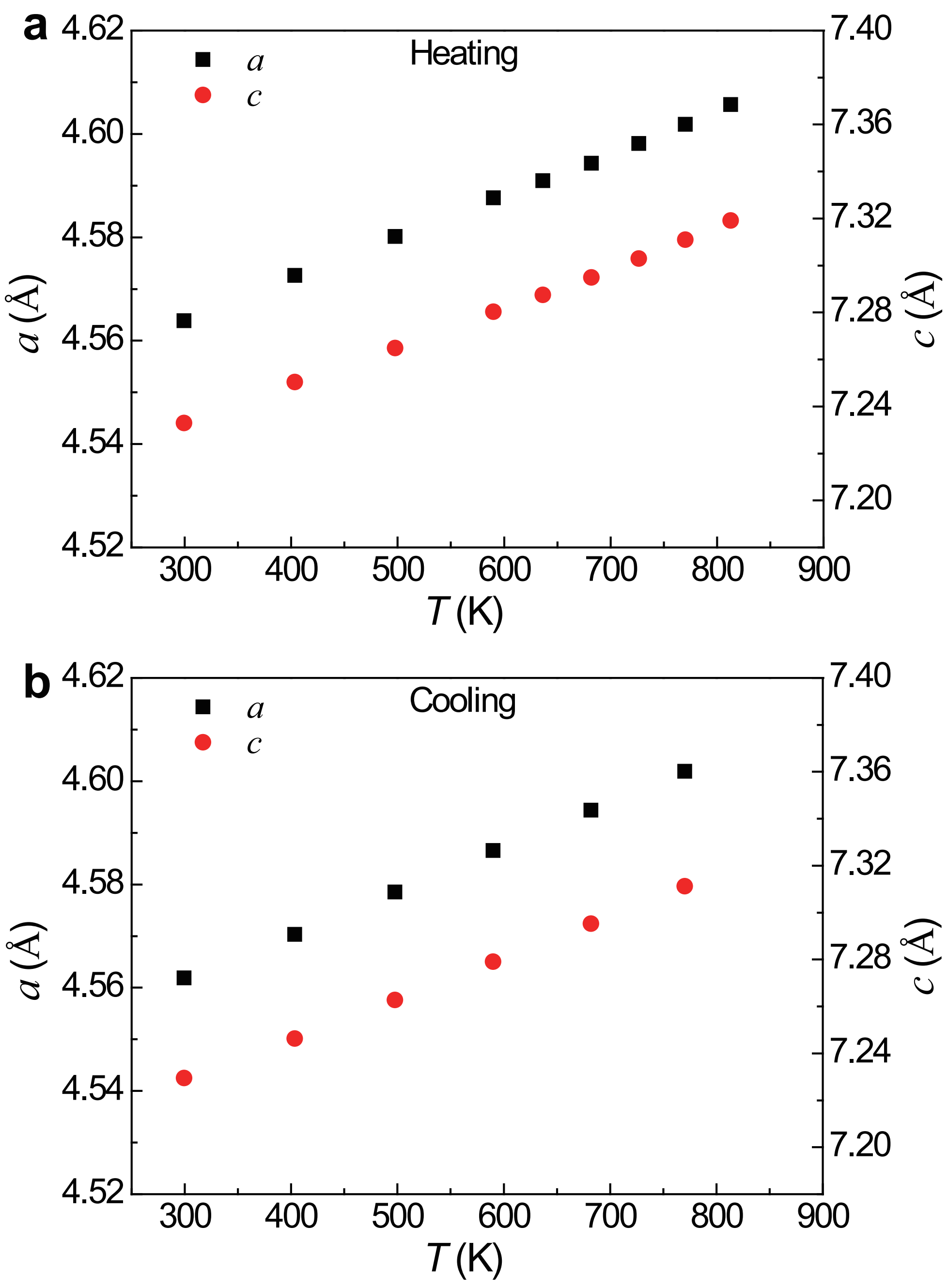

Supplementary Figure 5 | Temperature-dependent lattice parameters. Temperature dependence of lattice parameters of Mg_3Sb_2 for the temperature points of (a) 299-813 K and (b) 770-299 K. The uncertainty is smaller than the size of the symbol.

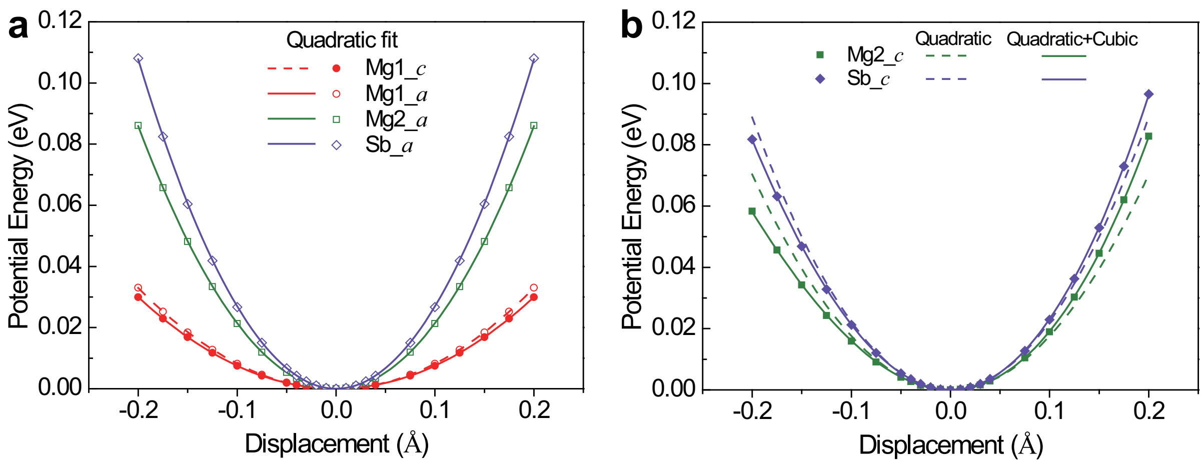

Supplementary Figure 6 | Fitted potential energy curves. Fitted potential energy curves for the nonequivalent atoms along the axial directions in Mg_3Sb_2. Potential wells of Mg1 along axial directions, Mg2 along the *a* direction, and Sb along the *a* direction shown in (a) are harmonic, whereas potential wells of Mg2 and Sb along the *c* direction shown in (b) are anharmonic with cubic terms.

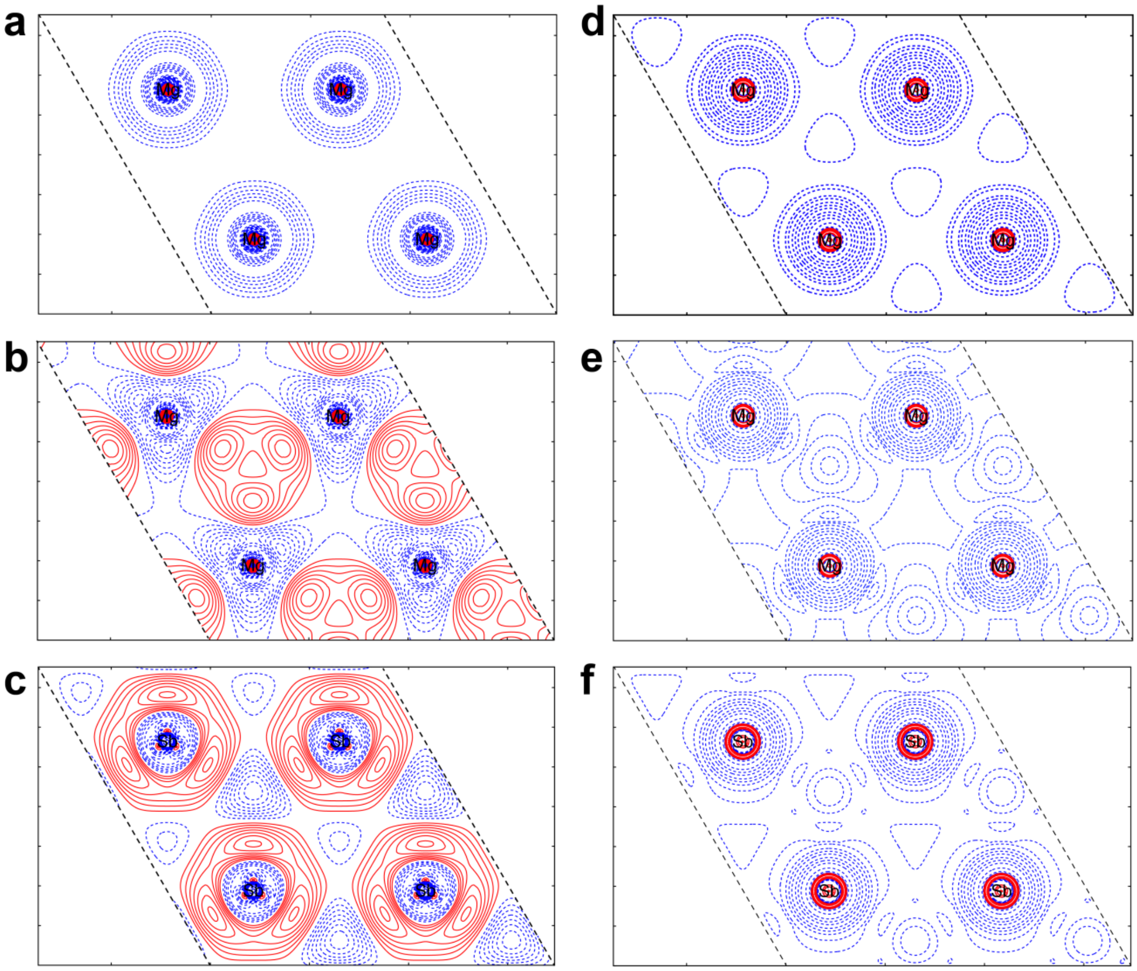

Supplementary Figure 7 | Static deformation density and negative Laplacian maps. (a-c) Static deformation maps on a-b planes containing (a) Mg1 atoms, (b) Mg2 atoms, and (c) Sb atoms of Mg_3Sb_2. The contour interval is 0.006 e/Å^3. **(d-f)** Negative Laplacian maps on a-b planes of (d) Mg1 atoms, (e) Mg2 atoms, and (f) Sb atoms of Mg_3Sb_2. Contours are plotted at $\pm 2\times10^n$, $\pm 4\times10^n$ and $\pm 8\times10^n$ e/Å^5 (n = ±3, ±2, ±1, 0). Red solid and blue dotted lines represent positive and negative values, respectively.

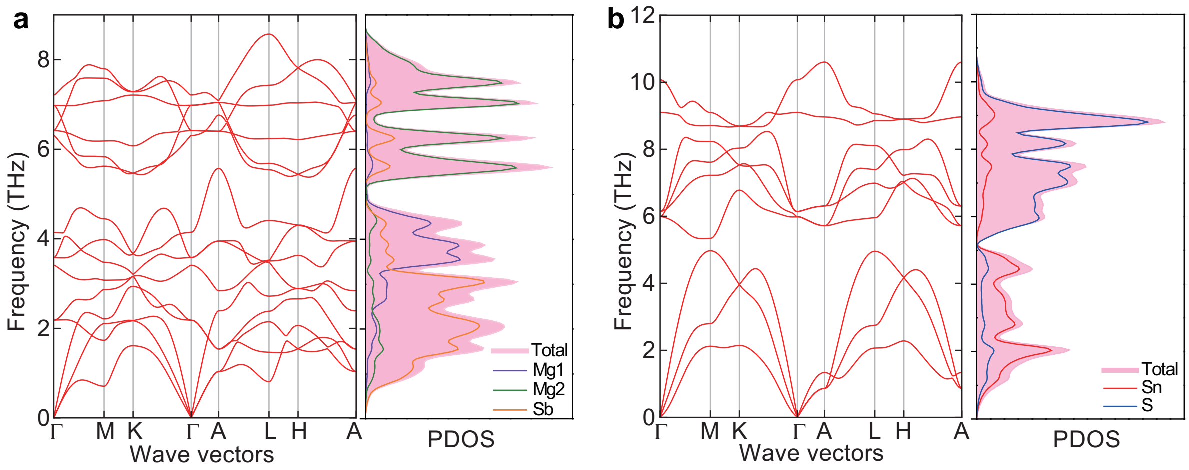

Supplementary Figure 8 | Phonon band structures and density of states. Phonon dispersions and phonon density of states (PDOS) of (a) Mg_3Sb_2 and (b) SnS_2.

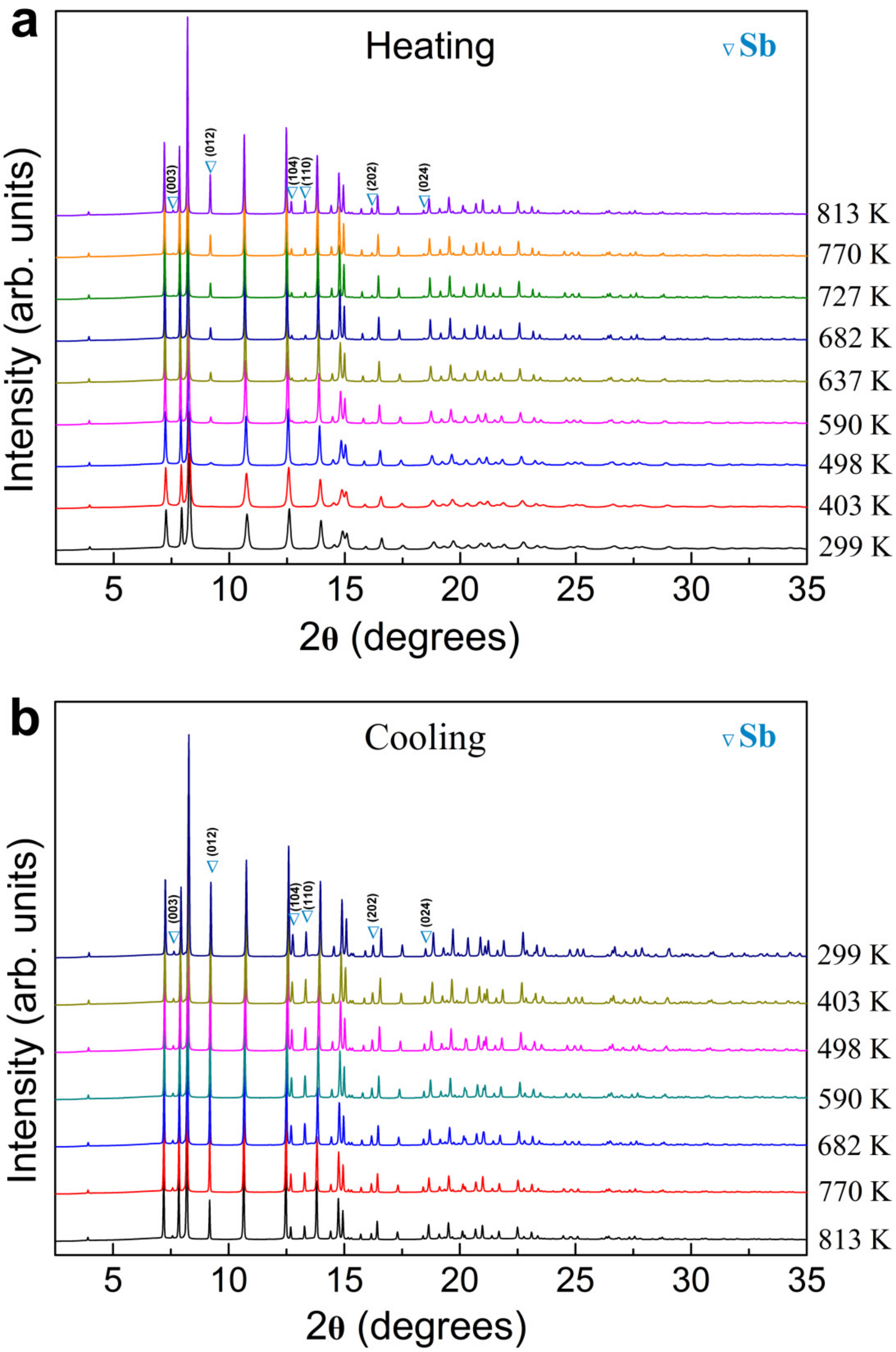

Supplementary Figure 9 | Multi-temperature synchrotron PXRD data. Multi-temperature synchrotron PXRD patterns of Mg_3Sb_2 at temperatures of (a) 299-813 K (heating) and 813-299 K (cooling).

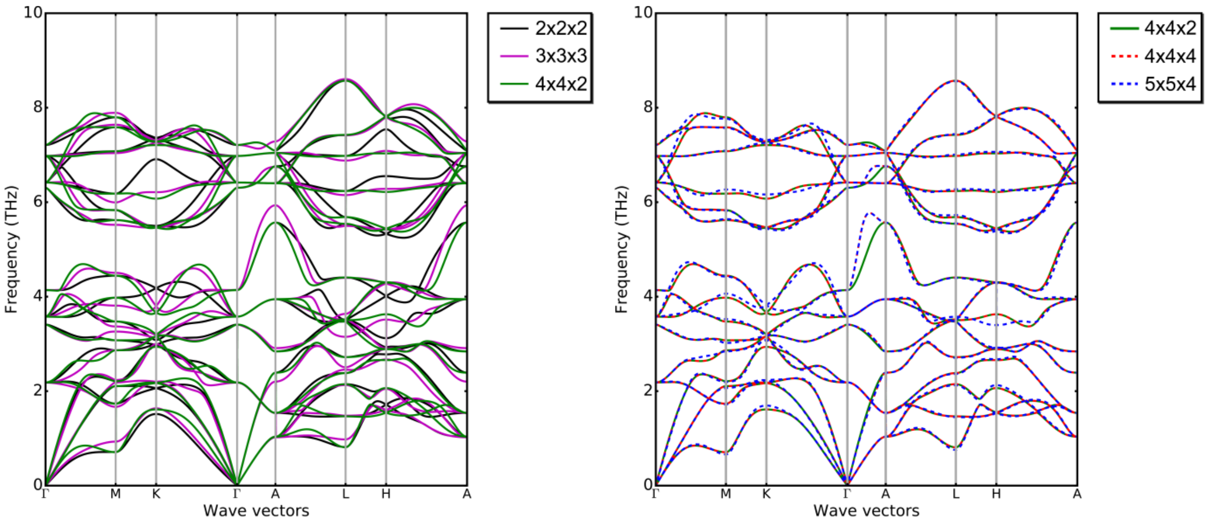

Supplementary Figure 10 | Convergence test of phonon dispersion. Convergence test of phonon dispersion of Mg_3Sb_2 with a series of supercell sizes. It is clear that, in order to ensure the well-converged phonon frequencies of acoustic branches, at least a supercell size of 4×4×2 (160 atoms) should be used. This reveals that the reported phonon-related results in ref. 1 based on a 2×2×2 supercell are simply not converged.

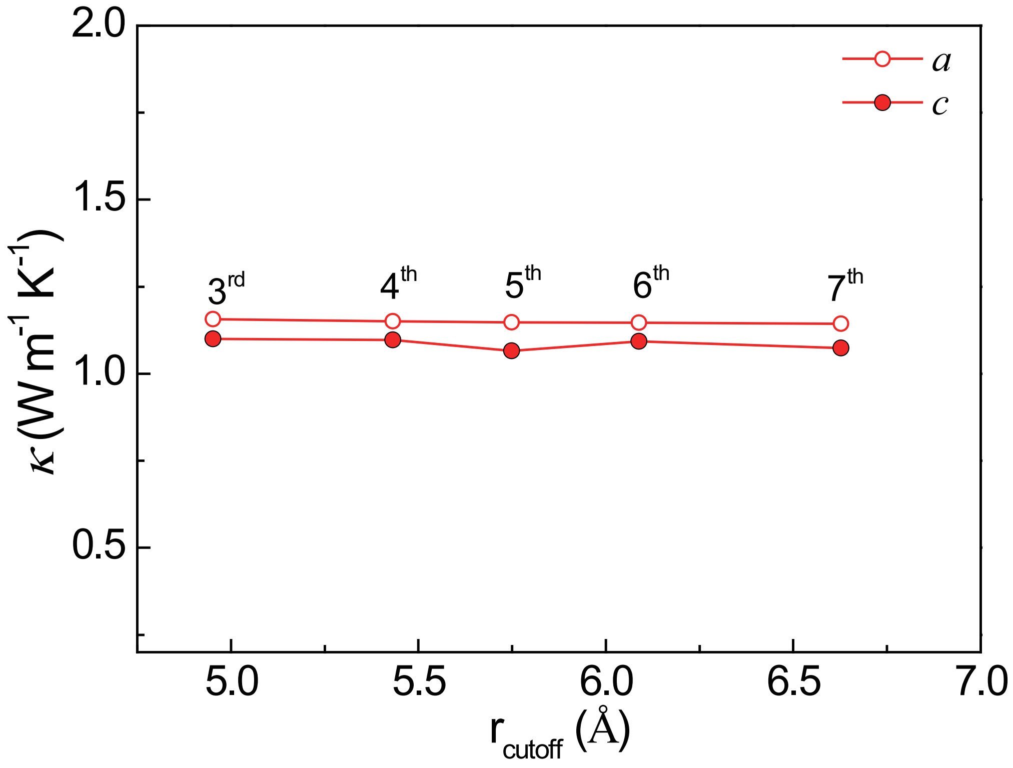

Supplementary Figure 11 | Convergence of the lattice thermal conductivity. Convergence of the lattice thermal conductivity of Mg_3Sb_2 with respect to the cutoff distance (r_{cutoff}) corresponding to the interaction range from the third to the seventh nearest neighbors for the anharmonic calculations.

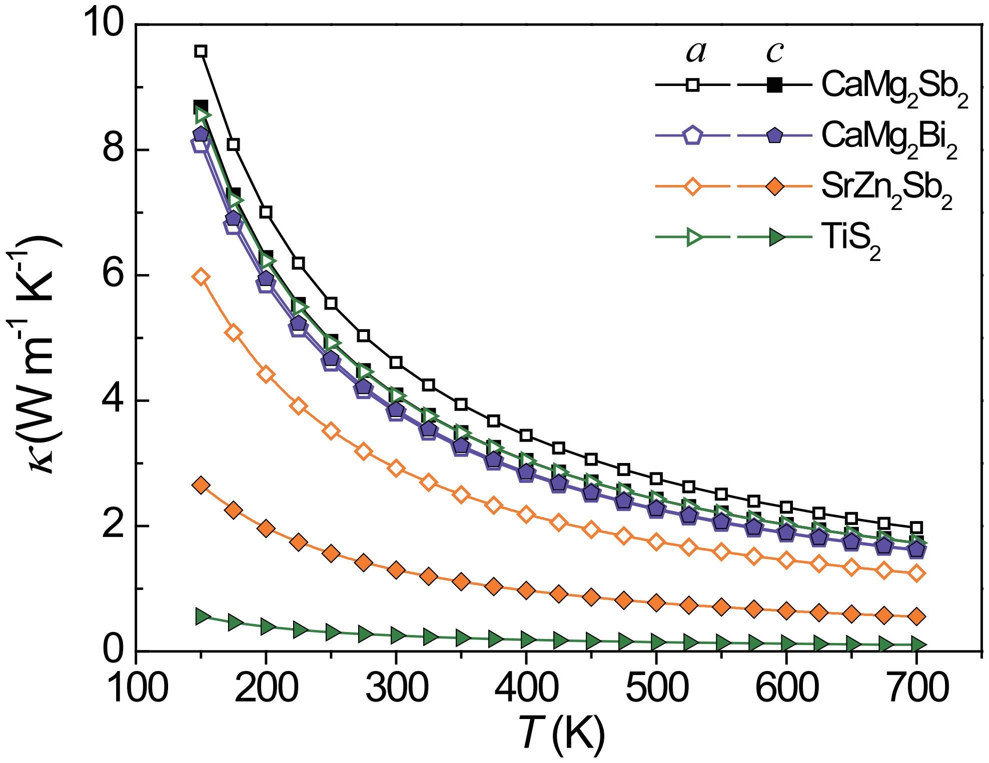

Supplementary Figure 12 | The theoretical lattice thermal conductivity. The calculated lattice thermal conductivity of TiS_2, $SrZn_2Sb_2$, $CaMg_2Sb_2$, and $CaMg_2Bi_2$ along the *a* and *c* directions.

Supplementary Tables

Supplementary Table 1 | Refinement details of synchrotron PXRD data upon cooling. Rietveld refinement details of the synchrotron PXRD data of Mg_3Sb_2 at 770-299 K upon cooling. The *R* factors and χ^2 shown here are the data from the Mg_3Sb_2 phase. T_{actual} represents the actual temperature calibrated by the thermocouple.

T_{actual}(K)		770.15	682.15	590.15	497.65	403.45	299.45
No. of points		7727	7727	7727	7727	7727	7728
No. of reflections		938	926	920	920	910	904
No. of parameters		48	48	48	48	48	48
R_F (%)		9.50	7.82	6.91	4.47	3.38	2.63
R_{Bragg} (%)		4.78	4.69	4.73	4.88	4.51	5.60
R_p (%)		9.78	9.45	9.61	9.86	8.94	8.54
R_{wp} (%)		10.6	10.5	10.7	11.3	10.6	10.2
χ^2		6.33	6.67	7.76	9.49	9.22	9.35
Wt.% Mg_3Sb_2		85.82(0.20)	85.24(0.19)	85.20(0.20)	85.23(0.21)	85.79(0.20)	85.53(0.19)
Wt.% Sb		14.18(0.06)	14.76(0.06)	14.80(0.06)	14.77(0.07)	14.21(0.06)	14.47(0.06)
Mg_3Sb_2	*a*=*b* (Å)	4.60190(5)	4.59434(5)	4.58658(4)	4.57851(4)	4.57029(4)	4.56187(3)
	c (Å)	7.31115(8)	7.29523(8)	7.27911(8)	7.26271(8)	7.24629(7)	7.22944(6)
Volume (Å^3)		134.088(3)	133.357(2)	132.613(2)	131.849(2)	131.079(2)	130.293(2)
U_{iso} (Å^2)	Mg1	0.05806(225)	0.05227(209)	0.04022(191)	0.02965(175)	0.02312(151)	0.01423(120)
	Mg2	0.03712(105)	0.03175(95)	0.02566(90)	0.01824(82)	0.01381(70)	0.00907(58)
	Sb	0.03404(23)	0.02877(22)	0.02338(19)	0.01792(18)	0.01374(14)	0.00876(11)
Occupancy	Mg1	0.07747(56)	0.07851(56)	0.07858(58)	0.07883(61)	0.07881(58)	0.07867(55)
	Mg2	0.15946(70)	0.16008(70)	0.16100(73)	0.16204(78)	0.16294(74)	0.16365(71)
	Sb	0.16667	0.16667	0.16667	0.16667	0.16667	0.16667

Supplementary Table 2 | Thermal expansion coefficients calculated by the cooling data. Lattice parameters *a* and c as a function of temperature and thermal expansion coefficients α_a and α_c at 299.45 K of Mg_3Sb_2. The data at 770-299 K upon cooling are used.

Sample	*a* (Å)	α_a ($\times10^{-5}$ K^{-1}) at 299.45 K	*c* (Å)	α_c ($\times10^{-5}$ K^{-1}) at 299.45 K	α_c/α_a at 299.45 K
Mg_3Sb_2	(1st degree) $a = 4.5360(2) + 8.56(4))\times10^{-5}T$	1.88(0)	(1st degree) $c = 7.1761(8) + 17.5(1)\times10^{-5}T$	2.42(1)	1.29(0)
	(2nd degree) $a = 4.5369(7) + 8.2(3)\times10^{-5}T + 0.3(2)\times10^{-8}T^2$	1.84(4)	(2nd degree) $c = 7.182(1) + 15.3(4)\times10^{-5}T + 2.0(3)\times10^{-8}T^2$	2.28(3)	1.24(3)

Supplementary Table 3 | Group velocities of the acoustic phonon branches. Group velocities of the three acoustic phonon branches along the Γ-M and Γ-A wave vector path in Mg_3Sb_2 and SnS_2. Γ-M and Γ-A correspond to the a^* and c^* directions, respectively.

	Mg_3Sb_2		SnS_2	
Wave vector path	Γ-M	Γ-A	Γ-M	Γ-A
TA1 group velocity (m s^{-1})	1969	1885	1149	1538
TA2 group velocity (m s^{-1})	2021	1885	2671	1538
LA group velocity (m s^{-1})	4340	4732	5297	2444

Supplementary Table 4 | Topological properties of the bond critical points. Topological properties of the bond critical points (**r**$_b$) in several Mg-containing compounds with $CaAl_2Si_2$-type structure.

Bond	d (Å)	$\rho(\mathbf{r}_b)$ (e Å^{-3})	$\nabla^2\rho(\mathbf{r}_b)$ (e Å^{-5})	G (a.u.)	V (a.u.)	H (a.u.)	$\|V\|/G$	G/ρ (a.u.)
				Mg_3Bi_2				
Tilted Mg2-Bi	2.953	0.152	0.953	0.0118	-0.0136	-0.0019	1.160	0.521
Vertical Mg2-Bi	3.040	0.144	0.761	0.0100	-0.0121	-0.0021	1.209	0.468
Interlayer Mg1-Bi	3.176	0.107	0.516	0.0064	-0.0075	-0.0011	1.167	0.407
				$CaMg_2Sb_2$				
Tilted Mg-Sb	2.868	0.167	1.243	0.0146	-0.0164	-0.0018	1.120	0.591
Vertical Mg-Sb	2.934	0.156	1.070	0.0128	-0.0144	-0.0017	1.130	0.554
Interlayer Ca-Sb	3.278	0.116	0.781	0.0087	-0.0092	-0.0006	1.065	0.506
				$CaMg_2Bi_2$				
Tilted Mg-Bi	2.954	0.153	0.993	0.0121	-0.0139	-0.0018	1.149	0.533
Vertical Mg-Bi	3.009	0.146	0.860	0.0108	-0.0126	-0.0019	1.173	0.498
Interlayer Ca-Bi	3.332	0.109	0.719	0.0080	-0.0084	-0.0005	1.062	0.491
				$SrMg_2Sb_2$				
Tilted Mg-Sb	2.891	0.164	1.175	0.0140	-0.0157	-0.0018	1.127	0.576
Vertical Mg-Sb	2.926	0.155	1.091	0.0129	-0.0145	-0.0016	1.122	0.560
Interlayer Sr-Sb	3.407	0.114	0.726	0.0082	-0.0089	-0.0007	1.082	0.486
				$YbMg_2Sb_2$				
Tilted Mg-Sb	2.864	0.167	1.254	0.0147	-0.0164	-0.0017	1.115	0.595
Vertical Mg-Sb	2.939	0.155	1.052	0.0126	-0.0143	-0.0017	1.135	0.549
Interlayer Yb-Sb	3.259	0.148	0.888	0.0111	-0.0130	-0.0019	1.170	0.505

Supplementary Table 5 | Topological properties of the additional bond critical point. Topological properties of the additional bond critical point found between interlayer Sb atoms. Additional bond critical point of interlayer Sb-Sb interaction is very weak and might be caused by numerical errors.

Bond	d (Å)	$\rho(\mathbf{r}_b)$ (e Å^{-3})	$\nabla^2\rho(\mathbf{r}_b)$ (e Å^{-5})	G (a.u.)	V (a.u.)	H (a.u.)	$\|V\|/G$	G/ρ (a.u.)
				$CaZn_2Sb_2$				
Interlayer Sb-Sb	4.473	0.049	0.177	0.0020	-0.0022	-0.0002	1.082	0.277
				$CaMg_2Sb_2$				
Interlayer Sb-Sb	4.585	0.048	0.192	0.0021	-0.0022	-0.0001	1.049	0.292
				$CaMg_2Bi_2$				
Interlayer Bi-Bi	4.627	0.047	0.176	0.0020	-0.0021	-0.0001	1.065	0.279
				$SrMg_2Sb_2$				
Interlayer Sb-Sb	4.720	0.038	0.144	0.0015	-0.0015	-7.5×10^{-6}	1.005	0.269
				$YbMg_2Sb_2$				
Interlayer Sb-Sb	4.542	0.054	0.212	0.0024	-0.0026	-0.0002	1.078	0.298

Supplementary Table 6 | Atomic properties. Atomic charges *Q* and atomic basin volumes *V* of several Mg-containing compounds with $CaAl_2Si_2$-type structure.

Atoms	*Q* (e)	*V* (Å^3)
	Mg_3Bi_2	
Mg1	1.43	9.79
Mg2	1.40	9.81
Bi	-2.11	57.11
	$CaMg_2Sb_2$	
Ca	1.39	16.01
Mg	1.48	8.63
Sb	-2.17	55.53
	$CaMg_2Bi_2$	
Ca	1.37	16.65
Mg	1.42	9.67
Bi	-2.10	59.14
	$SrMg_2Sb_2$	
Sr	1.38	21.74
Mg	1.47	8.87
Sb	-2.16	57.29
	$YbMg_2Sb_2$	
Yb	1.34	19.84
Mg	1.47	8.57
Sb	-2.14	52.96

Supplementary Table 7 | Refinement details of synchrotron PXRD data upon heating. Rietveld refinement details of the synchrotron X-ray powder diffraction data at 299-813 K upon heating. The *R* factors and χ^2 shown here are the data from the Mg_3Sb_2 phase. T_{actual} represents the actual temperature calibrated by the thermocouple.

T_{actual} (K)		299.45	403.45	497.65	590.15	682.15	726.6	770.15	813.0
No. of points		7727	7727	7727	7727	7727	7727	7727	7727
No. of reflections		1055	1071	994	963	918	920	924	938
No. of parameters		46	45	48	48	48	48	48	48
R_F (%)		1.37	1.48	3.31	4.45	7.21	9.51	9.84	10.7
R_{Bragg} (%)		5.77	4.34	8.12	9.99	5.30	4.91	5.18	4.24
R_p (%)		8.23	8.77	9.88	11.0	8.54	8.65	9.05	8.69
R_{wp} (%)		10.7	11.2	12.0	12.2	8.92	8.94	9.22	9.05
χ^2		8.17	7.91	8.57	9.06	4.91	4.85	4.94	4.51
Wt.% Mg_3Sb_2		98.77(0.30)	98.72(0.24)	97.51(0.26)	97.28(0.25)	96.84(0.18)	96.32(0.18)	95.03(0.18)	90.80(0.17)
Wt.% Sb		1.23(0.19)	1.28(0.07)	2.49(0.06)	2.72(0.05)	3.16(0.03)	3.68(0.03)	4.97(0.03)	9.20(0.04)
Mg_3Sb_2	*a*=*b* (Å)	4.56386(8)	4.57260(8)	4.58017(7)	4.58760(6)	4.59432(3)	4.59811(3)	4.60187(4)	4.60569(4)
	c (Å)	7.23289(15)	7.25042(15)	7.26486(12)	7.28032(11)	7.29486(6)	7.30297(6)	7.31098(6)	7.31910(7)
Volume (Å^3)		130.469(4)	131.286(4)	131.984(4)	132.694(3)	133.349(2)	133.718(2)	134.083(2)	134.455(2)
U_{iso} (Å^2)	Mg1	0.00524(121)	0.00949(176)	0.01672(180)	0.02389(189)	0.04560(152)	0.04857(160)	0.05315(175)	0.05535(180)
	Mg2	0.00822(71)	0.01344(92)	0.02221(99)	0.02388(98)	0.03545(75)	0.03699(77)	0.03965(84)	0.04421(91)
	Sb	0.00767(18)	0.01147(22)	0.01782(22)	0.02311(23)	0.02914(15)	0.03132(16)	0.03402(18)	0.03779(19)
Occupancy	Mg1	0.07364(57)	0.07320(59)	0.07438(59)	0.07459(60)	0.07878(44)	0.07712(44)	0.07716(45)	0.07753(46)
	Mg2	0.16277(74)	0.16268(78)	0.16263(78)	0.16096(78)	0.16440(57)	0.16255(57)	0.16155(58)	0.15928(58)
	Sb	0.16667	0.16667	0.16667	0.16667	0.16667	0.16667	0.16667	0.16667

Supplementary Table 8 | Thermal expansion coefficients calculated by the heating data. Lattice parameters *a* and c as a function of temperature and thermal expansion coefficients α_a and α_c at 299.45 K of Mg_3Sb_2. The data at 299-810 K upon heating are adopted.

Sample	*a* (Å)	α_a ($\times10^{-5}$ K^{-1}) at 299.45 K	*c* (Å)	α_c ($\times10^{-5}$ K^{-1}) at 299.45 K	α_c/α_a at 299.45 K
Mg_3Sb_2	(1st degree) a = 4.5400(4) + 8.03(6))$\times10^{-5}T$	1.76(1)	(1st degree) c = 7.183(1) + 16.6(2)$\times10^{-5}T$	2.30(1)	1.31(1)
	(2nd degree) a = 4.540(1) + 7.9(5)$\times10^{-5}T$ + 0.1(4)$\times10^{-8}T^2$	1.74(7)	(2nd degree) c = 7.190(3) + 14(1)$\times10^{-5}T$ + 2.5(8)$\times10^{-8}T^2$	2.14(8)	1.23(5)

Supplementary Table 9 | Debye temperatures. Debye temperatures of Mg_3Sb_2 obtained from the fitting of U_{iso} with the Debye expression using the temperature points of 770-299 K upon cooling. The averaged values were estimated from fitting of averaged U_{iso} = 1/5 U_{iso}(Mg1) + 2/5 U_{iso}(Mg2) + 2/5 U_{iso}(Sb). The values of Debye temperature obtained by fitting using the Debye expression are compared with the value reported in the literature[2].

Compound	Atoms	Debye temperature Θ_D (K)	
		Debye expression (This work)	Calculated from elastic constants[2]
Mg_3Sb_2	Mg1	248(6)	-
	Mg2	311(7)	-
	Sb	149(2)	-
	Average	187(4)	223

Supplementary Table 10 | The average Grüneisen parameter by another method. The average Grüneisen parameter at 300 K along the *a* and *c* directions obtained using Equation 3.

Compounds	$\tilde{\gamma}_a$	$\tilde{\gamma}_c$	$\tilde{\gamma}_c / \tilde{\gamma}_a$
Mg_3Sb_2	2.1	2.6	1.2
SnS_2	2.0	3.7	1.9

Supplementary Notes

Supplementary Note 1. Topological analysis of electron density

The quantitative analysis of chemical bonding in this work is based on Bader's quantum theory of atoms in molecules (QTAIM)[3]. QTAIM is based on the analysis of critical points (CPs) of the electron density, which are defined as the points satisfying $\nabla\rho = 0$. In general, CPs are classified according to the *rank*, i.e., the number of nonzero eigenvalues of the Hessian matrix, and the *signature*, which is the sum of the signs of the Hessian eigenvalues. These two characteristics are then used to label the CPs (*rank*, *signature*)[3,4]. Accordingly, there are four types of rank 3 CPs: (3, –3), i.e., maxima or nuclear CPs (NCP or n); (3, –1), first-order saddle or bond (BCP or b); (3, +1), second-order saddle or ring (RCP or r); and (3, +3), minima or cage (CCP or c). The illustration of critical points in Mg_3Sb_2 is shown in Supplementary Figs. 1 and 2. An atom in a molecule or crystal is defined as the space with a density maximum surrounded by a zero-flux gradient surface *S*:[3,4]

$$\nabla\rho(\mathbf{r})\cdot\mathbf{n}(\mathbf{r}) = 0 \quad \forall\mathbf{r}\in S(\mathbf{r}_s)\,, \tag{1}$$

where $\mathbf{n}(\mathbf{r})$ is the a unit vector perpendicular to the surface *S* at $\mathbf{r}$. An atom is described as the union of an attractor and its basin. The atomic property of an atom is then calculated by the integration within its atomic basin.

Supplementary Note 2. Synchrotron PXRD patterns and Rietveld refinement

From multi-temperature synchrotron PXRD patterns of Mg_3Sb_2 shown in Supplementary Fig. 9, we can see that the Sb phase appears as the temperature increases. To keep consistency, the secondary phase Sb is included in the refinements of all temperature points. Although there is a small difference between the thermal expansion coefficients calculated by the data upon heating and cooling, the anisotropic ratio of thermal expansion coefficient upon heating and cooling is nearly the same (see Supplementary Tables 8 and 2). As shown in Supplementary Table 7, the amount of Sb phase is gradually increasing with increasing temperature from 299 K to 810 K, whereas the amount of Sb phase becomes very stable upon cooling from 770 K to 299 K (see Supplementary Table 1). To avoid the possible influence of the increasing Sb

phase upon heating, we thereby use the cooling data at 770-299 K with the stable amount of Sb in the main text. The underlying mechanism of the appearing Sb phase upon heating in Mg_3Sb_2 powder will be discussed in our future work.

The Debye temperature was extracted by fitting the isotropic atomic displacement parameters U_{iso} based on a Debye model:[5-7]

$$U_{\mathrm{iso}} = \frac{3\hbar^2 T}{mk_{\mathrm{B}}\Theta_{\mathrm{D}}^2}\left[\frac{T}{\Theta_{\mathrm{D}}}\int_0^{\frac{\Theta_{\mathrm{D}}}{T}} \frac{x}{e^x - 1}dx + \frac{\Theta_{\mathrm{D}}}{T}\right] + d^2, \quad (2)$$

where $\hbar$ is the reduced Planck constant, T the absolute temperature, m is the mass of the atom, k_{B} is the Boltzmann constant, Θ_{D} is the Debye temperature, and d is a disorder parameter[8]. The Debye temperatures of Mg_3Sb_2 obtained from the fitting of U_{iso} at 770-299 K (upon cooling) with the Debye expression are shown in Supplementary Table 2. The average Debye temperature obtained by fitting using the Debye expression is slightly lower than the reported theoretical value[2] calculated from elastic constants of Mg_3Sb_2.

Supplementary Note 3. Theoretical calculation

The Grüneisen parameters for the acoustic modes might be negative, which will lead to the cancellation between the acoustic and optical modes. In addition to the average method used in the main text, the average Grüneisen parameter is calculated using the sum over the squared Grüneisen parameter:[9]

$$\tilde{\gamma}^2 = \frac{\sum_{\mathbf{q},i}[\gamma(\mathbf{q},i)]^2 C_V(\mathbf{q},i)}{\sum_{\mathbf{q},i} C_V(\mathbf{q},i)}, \quad (3)$$

where $\gamma(\mathbf{q},i)$ and $C_V(\mathbf{q},i)$ are the mode Grüneisen parameter and mode heat capacity for the phonon branch i at wave vector $\mathbf{q}$, respectively. The average Grüneisen parameters along the axial directions were calculated by summing over all phonon modes along the corresponding directions and the result is shown in Supplementary Table 10.

The lattice thermal conductivity of TiS_2 was calculated by ShengBTE code[10] based on a full iterative solution to the Boltzmann transport equation for phonons. The second-order and

third-order interatomic force constants were computed in the 4×4×2 supercells. The displacement amplitude of 0.09 Å was adopted for harmonic force constants calculations to ensure the well-converged properties[11]. Van der Waals functional optB86b-vdW[12] in VASP[13] code was used for all calculations with an energy convergence criterion of 10^{-6} eV and a plane wave energy cutoff of 600 eV.

Supplementary Reference

1. Tamaki, H., Sato, H. K. & Kanno, T. Isotropic conduction network and defect chemistry in $Mg_{3+\delta}Sb_2$-based layered Zintl compounds with high thermoelectric performance. *Adv. Mater.* **28,** 10182-10187 (2016).
2. Tani, J.-i., Takahashi, M. & Kido, H. Lattice dynamics and elastic properties of Mg_3As_2 and Mg_3Sb_2 compounds from first-principles calculations. *Physica B* **405,** 4219-4225 (2010).
3. Bader, R. F. W. *Atoms in Molecules: A Quantum Theory* (Oxford University Press, Oxford, 1990).
4. Gatti, C. Chemical bonding in crystals: new directions. *Z. Kristallogr.* **220,** 399-457 (2005).
5. Willis, B. T. M. & Pryor, A. W. *Thermal Vibrations in Crystallography* (Cambridge University Press, Cambridge, 1975).
6. Sales, B. C., Chakoumakos, B. C., Mandrus, D. & Sharp, J. W. Atomic displacement parameters and the lattice thermal conductivity of clathrate-like thermoelectric compounds. *J. Solid State Chem.* **146,** 528-532 (1999).
7. Blichfeld, A. B. & Iversen, B. B. Fast direct synthesis and compaction of phase pure thermoelectric ZnSb. *J. Mater. Chem. C* **3,** 10543-10553 (2015).
8. Bentien, A., Nishibori, E., Paschen, S. & Iversen, B. B. Crystal structures, atomic vibration, and disorder of the type-I thermoelectric clathrates $Ba_8Ga_{16}Si_{30}$, $Ba_8Ga_{16}Ge_{30}$, $Ba_8In_{16}Ge_{30}$, and $Sr_8Ga_{16}Ge_{30}$. *Phys. Rev. B* **71,** 144107 (2005).
9. Bjerg, L., Iversen, B. B. & Madsen, G. K. H. Modeling the thermal conductivities of the zinc antimonides ZnSb and Zn_4Sb_3. *Phys. Rev. B* **89,** 024304 (2014).

10. Li, W., Carrete, J., A. Katcho, N. & Mingo, N. ShengBTE: a solver of the Boltzmann transport equation for phonons. *Comput. Phys. Commun.* **185,** 1747-1758 (2014).
11. Lindroth, D. O. & Erhart, P. Thermal transport in van der Waals solids from first-principles calculations. *Phys. Rev. B* **94,** 115205 (2016).
12. Klimeš, J., Bowler, D. R. & Michaelides, A. Van der Waals density functionals applied to solids. *Phys. Rev. B* **83,** 195131 (2011).
13. Kresse, G. & Furthmüller, J. Efficient iterative schemes for ab initio total-energy calculations using a plane-wave basis set. *Phys. Rev. B* **54,** 11169-11186 (1996).
