# Supplementary material for: Chemical bonding origin of the unexpected isotropic physical properties in thermoelectric Mg3Sb2 and related materials
Source: Nat Commun. 2018 Nov 9;9:4716. doi: 10.1038/s41467-018-06980-x (PMC6226478; doi:10.1038/s41467-018-06980-x)
Supplement: Supplementary file 2 — Peer Review File [file 41467_2018_6980_MOESM2_ESM.pdf]

Reviewers' comments:

Reviewer #1 (Remarks to the Author):

I would like to provide my comments only on the concept and novelty of this work:
1.Anything causing an X-ray diffraction peak indicates an existence of atomic layer(s). By this definition, all crystalline solids, including cubic structures, can be called as a 'layered structure'. The bonding length between the so-called 'layers' is actually much shorter (and possibly stronger) than that in the 'layer(s)' for Mg_3Sb_2 and the related AB_2C_2 compounds, the reviewer would be interested in how easy the cleavage along the 'layer' would be in these compounds. The authors should clarify what are the scientific reason(s) to safely define these compounds as 'layered' and why strongly anisotropic properties are expected?
2.If the answer turns out to be 'these compounds can not be safely defined as layer structures', then the discussion on property anisotropy and make comparison to really Van der Waals layered SnS_2 would be much less meaningful than the authors thought.
3.This closely links to the main conclusion of this work. The main claim in the manuscript that the chemical bonding in these compounds are weakly anisotropic, could be simply expected (but not unexpected as claimed by the authors in the title), because the structure itself is weakly anisotropic (although the authors tend to call it as 'a layered structure'). This commonsensible finding is insufficiently novel for a high impact journal.

With the above mentioned vaguely defined 'layered structure' to these compounds in this work, nearly isotropic properties are really expected but not unexpected. Therefore, the reviewer is unable to recommend for publication in Nature Communications unfortunately.

However, considering the systematical study on chemical bonding included in this work, I do recommend for publication in a more specialized journal, after careful revisions regarding to the claims on 'layered structure' and 'unexpected isotropic properties' for less misleading to readers.

BTW, a recent publication (https://doi.org/10.1016/j.joule.2018.06.014) on the same material might be illuminating for the authors to revise their claims for this work. Somehow, there are conflicting points between these two works.

Reviewer #2 (Remarks to the Author):

The present manuscript discusses the surprisingly isotropic properties of layered compounds based on Mg_3Sb_2. Given the recent surge of interest in 2D materials beyond graphene, this is a timely topic for the broad readership of Nature Communications. Mg_3Sb_2 is of particular interest, since it shows remarkable thermoelectric properties. The authors of the present manuscript derive a parameter, which characterizes the anisotropy of the lattice thermal conductivity. This is an interesting and relevant accomplishment. Hence, I recommend acceptance of this article.

There are a few minor points the authors might want to consider.

a) While the authors make very clear arguments, they could sometimes be improved regarding their presentation.
b) For example, it would be nice to describe the Zintl formalism in two sentences. It is very common in chemistry, but less so in material science and physics. Hence it would help readers from these communities, if the Zintl mechanism is described very briefly.
c) Then I did not find a table with bond distances for the different atoms in the manuscript. Again, this would help to readers to test, if there is a clear bond length – bond strength correlation.
d) I am also wondering if the Bader analysis of bonding could be presented first, since it is much more common than the NCI analysis.
e) The average Grüneisen parameter along the a and c axes in Mg_3Sb_2 are both rather low (and similar). This raises the question how the low thermal conductivity in Mg_3Sb_2 is explained.

Reviewer 1

Comment 1

‘*1.Anything causing an X-ray diffraction peak indicates an existence of atomic layer(s). By this definition, all crystalline solids, including cubic structures, can be called as a ‘layered structure’. The bonding length bet-ween the so-called ‘layers’ is actually much shorter (and possibly stronger) than that in the ‘layer(s)’ for Mg_3Sb_2 and the related AB_2C_2 compounds, the reviewer would be interested in how easy the cleavage along the ‘lay-er’ would be in these compounds. The authors should clarify what are the scientific reason(s) to safely define these compounds as ‘layered’ and why strongly anisotropic properties are expected?*’

Reply

We thank the referee for this comment. We believe this is a misunder-standing.

The definition of the $CaAl_2Si_2$-type compounds as “layered” is based on the widely-accepted Zintl formalism by the earlier work of R. Hoffmann *et al* (*J. Am. Chem. Soc.* **108,** 1876-1884 (1986).). Nearly all previous works then described Mg_3Sb_2 and related AB_2X_2 compounds with the $CaAl_2Si_2$-type structure as layered Zintl phases by assuming that the ionic A catio-nic layers donate electrons to the covalent B_2X_2 anionic layers (e.g. *J. Am. Chem. Soc.* **108,** 1876-1884 (1986); *Adv. Funct. Mater.* **15,** 1860-1864 (2005); *Nat. Mater.* **7,** 105-114 (2008); *J. Comput. Chem.* **29,** 2144-2153 (2008); *J. Electron. Mater.***42**, 1307-1312 (2013); *J. Mater. Chem. A* **2**,

12311-12316 (2014); *Adv. Mater.* **28,** 10182-10187 (2016); *J. Mater. Chem. A* **5,** 9050-9059 (2017); *Mater. Today Phys.* **1,** 74-95, (2017); etc.). The clear distinction between the ionic and the covalent layers gives rise to the much weaker interlayer A-X bond with respect to the intralayer B-X bonds. This simple layered Zintl phase view has been widely adopted in previous works. According to this widely-accepted layered Zintl phase view, we would expect AB_2X_2 compounds with the $CaAl_2Si_2$-type structure to show anisotropic structural and thermal properties. We find that Zn-containing AB_2X_2 compounds (e.g. $CaZn_2Sb_2$ and $SrZn_2Sb_2$) indeed show anisotropic thermal conductivities with $\kappa_a/\kappa_c \approx 2$, but Mg_3Sb_2 and other Mg-containing AB_2X_2 compounds show nearly isotropic feature in structural or thermal properties, which is unexpected. Therefore, we think the word "unexpected" used in the title is reasonable. For the word "layered" in the title of the initial version of the manuscript, we mean "presumed layered" claimed by previous references. In the revised manuscript, we have modified the title to make it clear (see the summary of revision below).

Moreover, structural and thermal properties are closely linked to chemical bonding character and strength, which cannot simply be evaluated from the bond length (see the reply to comment 3 for details). Whether a material is a layered structure or a Zintl phase can only be safely assessed and defined based on the detailed analysis of chemical bonding. In layered materials, the interlayer interactions should be much weaker than the intralayer interactions. Based on the quantitative analysis of chemical bonding in this work, we understand that Zn-containing AB_2X_2 compound can be described as a layered Zintl phase due to the much weaker interlayer bonds with respect to the intralayer bonds, whereas Mg_3Sb_2 and related Mg-containing AB_2X_2 compounds cannot be described as layered Zintl phases due to the nearly isotropic 3D chemical bonding network, which is the origin of the nearly isotropic structural and thermal properties.

It appears that the reviewer believes that the origin of X-ray diffraction is "layers" in crystals (*"Anything causing an X-ray diffraction peak indicates an existence of atomic layer(s)"*. This is false. Lattice planes are arbitrary constructs and have nothing to do with the X-ray scattering process. Atoms in crystals do not necessarily lie on planes (layers). The common use of lattice planes (e.g. in Bragg's law) has nothing to do with layering of atoms in a crystal structure. They refer to a lattice and diffraction occurs because of the translational symmetry of a crystal. Think of protein crystals. They still diffract, yet the unit cell can contain thousands of atoms without any "layers".

To address the reviewer's concern and make the idea of our work clear, the following revisions have been conducted:

(a) The word "layered materials" in the title has been modified as "Mg_3Sb_2-related materials".

(b) Three sentences in the beginning of the abstract have been modified as: "The Mg_3Sb_2 structure is currently being intensely scrutinized due to its outstanding thermoelectric properties. Usually, it is described as a layered Zintl phase with a clear distinction between covalent $[Mg_2Sb_2]^{2-}$ layers and ionic Mg^{2+} layers. Based on the quantitative chemical bonding analysis, we unravel instead that Mg_3Sb_2 exhibits a nearly isotropic three-dimensional (3D) bonding network……"

(c) A few words have been added in one sentence on page 2: "……In general, AB_2X_2 with the $CaAl_2Si_2$-type structure including Mg_3Sb_2 is understood as a layered Zintl phase by assuming that the covalent B_2X_2 anionic layer receives the electrons donated by the ionic A cationic layer[10,18-20]."

(d) Several words have been added in the beginning of page 3: "The AB_2X_2 compounds with the $CaAl_2Si_2$-type structure are expected to show anisotropic properties due to the commonly accepted notion that the interlayer A-X interaction is much weaker than the intralayer covalent bonding in the B_2X_2 layer[18,20]."

(e) A few words have been added in the end of page 3: "……Such a unique bonding feature in Mg_3Sb_2 not only challenges the well-known Zintl formalism and the description as a layered structure, but also results in……".

(f) A few words are modified in the end of page 7: "……Based on the above results, the description of a layered Zintl phase holds true for $CaZn_2Sb_2$ since it meets the assumption that the ionic A^{2+} layer donates electrons to the covalent $[B_2X_2]^{2-}$ layer; however, this description is not applicable to Mg_3Sb_2 since the intralayer bonds in $[Mg_2Sb_2]^{2-}$ slabs are not really covalent. ……".

(g) Several words have been added in the end of page 13: "……This result not only indicates that Mg_3Sb_2 cannot be described as a layered structure, but also challenges the widely accepted Zintl concept that assumes the $[Mg_2Sb_2]^{2-}$ slabs being covalently bonded. ……".

<u>Comment 2</u>

'2.If the answer turns out to be 'these compounds can not be safely defined as layer structures', then the discussion on property anisotropy and

make comparison to really Van der Waals layered SnS_2 would be much less meaningful than the authors thought.'

Reply

Regarding the "layered structure", please see the reply to Comment 1. Moreover, here we would like to emphasize that the reason of making comparison to van der Waals layered SnS_2 is that they show many structural similarities with AB_2X_2 compounds, which is clearly stated in our manuscript. Without considering the difference in lattice parameters, AB_2X_2 can be viewed as intercalating two monolayers of B ions into the van der Waals gap of SnS_2 and replacing Sn and S respectively by A and X. By comparing to the 2D van der Waals layered solid with the weak interlayer interaction being van der Waals force, we will have a better understanding of relatively stronger interlayer interaction as well as nearly isotropic 3D chemical bonding network in Mg-containing AB_2X_2 compounds. This gives rise to an important conclusion in our paper that the Mg-containing AB_2X_2 compounds cannot be described as layered materials, which, however, are claimed by many previous references (e.g. *Adv. Mater.* **28,** 10182-10187 (2016); *J. Mater. Chem. A* **5,** 9050-9059 (2017); *Mater. Today Phys.* **1,** 74-95, (2017).). Therefore, the comparison to 2D van der Waals MX_2 is very meaningful.

Comment 3

'*3.This closely links to the main conclusion of this work. The main claim in the manuscript that the chemical bonding in these compounds are weakly anisotropic, could be simply expected (but not unexpected as claimed by the authors in the title), because the structure itself is weakly anisotropic (although the authors tend to call it as 'a layered structure'). This commonsensible finding is insufficiently novel for a high impact journal.'*

Reply

We are sorry for this confusion. Below we explain why the conclusion in our paper is not common sensible.

Structural and phonon-related properties are closely linked to chemical bonding character and strength, which cannot be evaluated simply from bond lengths. Information based on bond length does not give us any hint on the type and ionicity of chemical bonding. The judgement from the bond length is oversimplified and can be problematic. How small should the difference between the three chemical bonds be to expect similar bonding covalency and nearly isotropic thermal properties? If this can be simply expected, then why did all previous references still claim that

Mg_3Sb_2 is a layered Zintl phase with the cationic Mg^{2+} layer being ionic and the $[Mg_2Sb_2]^{2-}$ slabs being covalently bonded? Bond lengths of Mg_3Sb_2 and other Mg-containing AB_2X_2 compounds have been reported and discussed in previous references (e.g. *J. Am. Chem. Soc.* **108,** 1876-1884 (1986); *Adv. Mater.* **28,** 10182-10187 (2016); *J. Mater. Chem. A* **5,** 9050-9059 (2017); etc.), whereas these studies still claimed Mg_3Sb_2 and related compounds as layered Zintl phases with the A layers being ionic and B_2X_2 layers being covalently bonded. This is a clear indication that simply from bond length we are unable to understand chemical bonding character and related properties. Therefore, the main conclusion in our paper cannot be simply expected.

Moreover, besides Mg_3Sb_2 coincidentally with a relatively small bond length difference between the three chemical bonds, many other Mg-containing $CaAl_2Si_2$-type compounds show relatively large bond length difference comparable to that of $CaZn_2Sb_2$ (see Table A). According to the reviewer's opinion, we would expect these compounds to show an obvious difference of covalency between interlayer and intralayer bonds as well as a large anisotropy in lattice thermal conductivity as that of $CaZn_2Sb_2$ (κ_a/κ_c=2.3). However, these compounds still show nearly isotropic 3D chemical bonding network with largely ionic chemical bonds, which result in nearly isotropic lattice thermal conductivity (e.g. $\kappa_a/\kappa_c \approx 1$ for $CaMg_2Sb_2$ and $CaMg_2Bi_2$). Therefore, the nearly isotropic thermal properties cannot simply be expected from bond length, and detailed quantitative analysis of chemical bonding is needed to understand chemical bonding and the related structural and thermal properties.

Based on the above points, it is clear that our work is definitely not a common sensible finding but a very novel discovery over previous works.

Table A. Bond lengths of several Mg-containing AB_2X_2 and $CaZn_2Sb_2$

Compounds	d (Å)		
	Interlayer A-X	Vertical B-X	Tilted B-X
Mg_3Sb_2	3.120	2.959	2.849
$CaMg_2Sb_2$	3.278	2.934	2.868
$CaMg_2Bi_2$	3.332	3.009	2.954
$SrMg_2Sb_2$	3.407	2.926	2.891
$YbMg_2Sb_2$	3.259	2.939	2.864
$CaZn_2Sb_2$	3.230	2.820	2.719

Comment 4

'*With the above mentioned vaguely defined 'layered structure' to these compounds in this work, nearly isotropic properties are really expected*

but not unexpected. Therefore, the reviewer is unable to recommend for publication in Nature Communications unfortunately.

However, considering the systematical study on chemical bonding included in this work, I do recommend for publication in a more specialized journal, after careful revisions regarding to the claims on 'layered structure' and 'unexpected isotropic properties' for less misleading to readers.'

<u>Reply</u>

Nearly isotropic properties cannot simply be expected and the conclusion in our paper is not common sensible (please see the reply to comment 3 for details). Furthermore, here we would like to point out that the novelty of a paper should be evaluated from the scientific content rather than simply assessed from the title. The novelty of the scientific content of our work is listed as follows:

(a) All earlier works have described Mg_3Sb_2 as a layered Zintl phase by assuming a clear existence of the covalent and ionic parts. However, here we reveal instead that the chemical bonds in Mg_3Sb_2 are all largely ionic, suggesting the breakdown of the widely-known Zintl formalism. This directly pinpoints that the simple layered structural views often used in this material are problematic. Earlier works on chemical bonding of the $CaAl_2Si_2$-type compounds using crystal orbital approaches are based on the concepts of $[Al_2Si_2]^{2-}$ networks (e.g. Zheng, et al. *J. Am. Chem. Soc.* **1986**, 108**,** 1876.), and the interlayer interactions are usually overlooked. We show that the interlayer and intralayer bonds are surprisingly similar in Mg_3Sb_2, which clearly means that the interlayer interaction cannot be neglected. Overall, our findings substantially reshape and extend our knowledge of chemical bonding in $CaAl_2Si_2$-type thermoelectric materials.

(b) There has never been any study providing a quantitative relationship between topological properties of chemical bonding and lattice thermal conductivity. Here we discover a nearly linear correlation between a parameter based on topological properties of chemical bonding and the anisotropy of lattice thermal conductivity in Mg_3Sb_2-related materials. This chemical bonding parameter can potentially be used to search for new presumed layered materials with tailored anisotropic lattice thermal conductivities.

(c) No studies so far have reported on the experimental lattice thermal expansion, temperature-dependent atomic displacement parameters, and lattice compression under pressure in Mg_3Sb_2, which are all properties having a strong bearing on the unexpected isotropic behavior. These physical properties as well as lattice thermal conductivity are

ultimately determined by the specific nature of the chemical bonding, and it is therefore essential to carry out a state of the art investigation of chemical bonding based on proper quantum mechanics, since this has not been attempted previously.

(d) The nature of the chemical bonding in a material determines its structural and thermal properties, but the specific relationship between bonding and properties is often very obscure. Based on the quantitative analysis of chemical bonding within the quantum theory of atoms in molecules, we unveil the nearly isotropic 3D chemical bonding network in Mg_3Sb_2, and this paves the way to understand the isotropic features in structural and thermal properties.

(e) Moreover, in our paper we demonstrate that the nearly isotropic 3D chemical bonding feature is not limited to Mg_3Sb_2, but can be broadly applied to other Mg-containing compounds with the $CaAl_2Si_2$-type structure. This indicates that the fundamental idea of understanding structure and thermal properties based on chemical bonding analysis generally can be extended to other related structures.

Based on the above points, we believe that the scientific content in our work represents a very significant advance over the earlier studies in terms of both novelty and fundamental insight into chemical bonding and properties of Mg_3Sb_2 and related $CaAl_2Si_2$-type compounds. It provides a shining example of how the nature of chemical bonding in a material determines its structural and thermal properties, and it shows that proper characterization of chemical bonding can lead to new concepts that allow the effective design of novel thermoelectric materials.

<u>Comment 5</u>

'*BTW, a recent publication (https://doi.org/10.1016/j.joule.2018.06.014) on the same material might be illuminating for the authors to revise their claims for this work. Somehow, there are conflicting points between these two works.*'

<u>Reply</u>

The recent publication (https://doi.org/10.1016/j.joule.2018.06.014) is focused on the possible explanation for the low thermal conductivity in Mg_3Sb_2, whereas our work is focused on the detailed chemical bonding analysis and reveals the origin of the isotropic feature in structural and thermal properties of Mg_3Sb_2. Overall, we cannot see a relation between two specific works since they are focused on different scientific topics. Moreover, our comprehensive, quantitative chemical bonding analysis

will provide the guidance for the simple, qualitative discussion of chemical bonding in this publication. It should be noted that this recent publication suggested by the reviewer also emphasizes Mg_3Sb_2 as a simple “layered” structure, which is conflicting with the aforementioned point from the reviewer.

Reviewer 2

Comment 1

‘*The present manuscript discusses the surprisingly isotropic properties of layered compounds based on Mg_3Sb_2. Given the recent surge of interest in 2D materials beyond graphene, this is a timely topic for the broad readership of Nature Communications. Mg_3Sb_2 is of particular interest, since it shows remarkable thermoelectric properties. The authors of the present manuscript derive a parameter, which characterizes the anisotropy of the lattice thermal conductivity. This is an interesting and relevant accomplishment. Hence, I recommend acceptance of this article.*’

Reply

We thank the referee for the positive comment.

Comment 2

‘*a) While the authors make very clear arguments, they could sometimes be improved regarding their presentation.*

b) For example, it would be nice to describe the Zintl formalism in two sentences. It is very common in chemistry, but less so in material science and physics. Hence it would help readers from these communities, if the Zintl mechanism is described very briefly.’

Reply

Thank you for the suggestion. We have added two sentences briefly describing the Zintl formalism in the $CaAl_2Si_2$-type materials. The corresponding revision is summarized below:

Two sentences regarding Zintl formalism are added in the end of page 2: “Zintl phases are charge-balanced compounds consisting of both covalently and ionically bonded atoms, where the ionic cations are considered as electron donors, donating electrons to the covalently-bonded anionic substructures. The covalent bonding in the anionic substructures ensures high carrier mobility, while the ionic cations allow the carrier density ma-

nipulation via doping without affecting the covalently bound network.[10,19]"

Comment 3

'c) Then I did not find a table with bond distances for the different atoms in the manuscript. Again, this would help to readers to test, if there is a clear bond length – bond strength correlation.'

Reply

Thank you for the comment. The bond distances for different atoms are listed in the second column of Table 1.

Comment 4

'd) I am also wondering if the Bader analysis of bonding could be presented first, since it is much more common than the NCI analysis.'

Reply

Thank you for the good suggestion. We have made the corresponding revision by moving the NCI analysis after the Bader analysis of bonding. The main revision is summarized below:

(a) The two paragraphs describing the NCI analysis are moved to the page 8, which is right after the discussion of the Bader atomic properties.

(b) Four panels of Figure 1 regarding NCI plots are removed and added in the revised manuscript as a new figure, i.e., Figure 2. The captions of all figures are updated. The coordination environments of all none-quivalent atoms are added in Figure 1c,d.

(c) The figure numbers in the main text and the order of the references are updated.

Comment 5

'e) The average Grüneisen parameter along the a and c axes in Mg_3Sb_2 are both rather low (and similar). This raises the question how the low thermal conductivity in Mg_3Sb_2 is explained.'

Reply

The average Grüneisen parameters of Mg_3Sb_2 along the *a* and *c* axes are 1.8 and 2.2, respectively, which are moderate and in the common range of ≥1.5 for materials with low thermal conductivity. The Grüneisen para-

meters of Mg_3Sb_2 are comparable to many state-of-the-art low thermal conductivity materials, for example, Bi_2Se_3 (1.65), Cu_3SbSe_3 (1.5), and $AgSbTe_2$ (2.05).

Summary of the changes (highlighted in the revised manuscript)

1. A few words of the title are modified.
2. Three sentences are modified in the beginning of the abstract. Several words in the abstract are modified. The number of words of the abstract is reduced to ~150 words.
3. Section headings and subheadings are added.
4. On page 2: one words is added in the end of the first paragraph; a few words are added in line 1, 4, and 9-11 of the second paragraph; two sentences are added in the end of the second paragraph.
5. On page 3: one word is added in line 2; several words are added in line 3; a few words are added in the end of the page.
6. On page 4: a few words are added in line 2, 6, and 7.
7. Four panels of Figure 1 are removed and added as a new figure, i.e., Figure 2. The captions of all figures are updated. Three small panels are added in Figure 1c,d. All figure notions in the main text are updated.
8. Two paragraphs on page 5 are moved to the page 8. The references order is updated.
9. On page 7: one sentence is removed in the end of the first paragraph; several words are modified in line 7-9 of the third paragraph.
10. On page 8: a few words are modified in line 4.
11. On page 9: a few words are modified in line 1 and 10.
12. On page 13: one word is added in line 4 of the first paragraph; a few words are modified in the end of the second paragraph; several words are added in the end of the page.
13. On page 14: one word is removed in the end of the first paragraph.
14. On page 15: one sentence is added in line 6 of the second paragraph.
15. A few words are added in the end of page 17.
16. On page 18: a few words are added in line 1 and 2.
17. “Data availability” statement is added on page 18 before the Reference section.

18. On page 19: the page number of reference 16 is updated.

19. On page 20: the order of the references 27-29 is updated.

20. The title in Supporting Information is updated.

Once again we would like to thank the reviewers for the time they have devoted to our manuscript. Their insightful comments have significantly improved our manuscript and we look much forward to your decision.

Sincerely yours,

Bo Brummerstedt Iversen

REVIEWERS' COMMENTS:

Reviewer #1 (Remarks to the Author):

I have gone through the authors’ response to the comments raised by the reviewers, and it seems that the authors address some of the comments technically. The authors better clarified their claims in the revised manuscript, mostly by rewording on ‘layered structure’. The reviewer does not think such a rewording adds sufficient significance to this work and the current findings are exciting enough.

Any claims on ‘layered structure’ or ‘unexpected isotropic properties’ in Mg_3Sb_2 and its related AMg_2X_2 compounds are misleading to the field. Common wisdom in this field is that isotropic 3D chemical bonding network would lead to isotropic properties. It is not a surprise to see a nearly isotropic bonding network in Mg_3Sb_2 and its related AMg_2X_2 compounds due to the structural features, therefore nearly isotropic phonon transport properties in these materials can be expected.

In order to improve the quality of this work, the authors might think to focus on the direct origin of 3D isotropic bonding network in these materials as a fundamental study for a more specific journal, which would definitely be highly appreciated by the field because thermoelectric Mg_3Sb_2 is now a rising star.

Reviewer #2 (Remarks to the Author):

The authors have convincingly addressed all my comments as well as the comments from the second referee. The detailed and clear response as well as the changes made are appreciated. I hence suggest publication without any further changes.

Regarding: Revised manuscript NCOMMS-18-17505B

Dear Dr. Cephas Small,

Thank you for the two referees' reports. We are grateful for their constructive and helpful comments, and below we address the reports point by point.

Reviewer 1

Comment 1

'*I have gone through the authors' response to the comments raised by the reviewers, and it seems that the authors address some of the comments technically. The authors better clarified their claims in the revised manuscript, mostly by rewording on 'layered structure'. The reviewer does not think such a rewording adds sufficient significance to this work and the current findings are exciting enough.*

Any claims on 'layered structure' or 'unexpected isotropic properties' in Mg_3Sb_2 and its related AMg_2X_2 compounds are misleading to the field. Common wisdom in this field is that isotropic 3D chemical bonding network would lead to isotropic properties. It is not a surprise to see a nearly isotropic bonding network in Mg_3Sb_2 and its related AMg_2X_2 compounds due to the structural features, therefore nearly isotropic phonon transport properties in these materials can be expected.

In order to improve the quality of this work, the authors might think to focus on the direct origin of 3D isotropic bonding network in these materials as a fundamental study for a more specific journal, which would definitely be highly appreciated by the field because thermoelectric Mg_3Sb_2 is now a rising star.'

Reply

It seems to us that this reviewer completely ignores our previous detailed response, indicating that this reviewer does not understand the concept of chemical bonding, which is the essential part of our work.

Chemical bonding character and strength cannot be evaluated simply from the structural features (i.e. bond lengths). Information based on bond length does not give us any hint on the type and ionicity of chemical bonding. How small should the difference between the three chemical bonds be to expect similar bonding covalency and nearly isotropic thermal properties? In addition, we have report that several AMg_2Sb_2 compounds with relatively large bond length differences comparable to those of AZn_2Sb_2 still show nearly isotropic lattice thermal conductivity, a clear confirmation of the point that nearly isotropic 3D bonding network cannot simply be expected from structural features. Please refer to the previous response letter for details.

Moreover, this reviewer keeps assessing the novelty from the title. However, we would like to emphasize that one should not evaluate novelty simply from a title, but from the scientific content with rational scientific arguments. The novelty of the scientific content of our paper was demonstrated in great detail in the previous response letter.

In the end of the comment, the reviewer believes the quality of a work can be improved by publishing in a more specific journal. This is obviously unreasonable. The quality of a work is dependent on the scientific content, and not where it is published. Overall, this reviewer does not provide unbiased comments with rational scientific arguments.

Reviewer 2

Comment 1

‘*The authors have convincingly addressed all my comments as well as the comments from the second referee. The detailed and clear response as well as the changes made are appreciated. I hence suggest publication without any further changes.*’

<u>Reply</u>

Thank you very much for carefully evaluating our response letter and providing positive comment. Moreover, we would like to express our sincere gratitude to you for providing the unbiased assessment on our response to the other reviewer’s comment.

Once again we would like to thank the reviewers for the time they have devoted to our manuscript. Their very insightful comments have significantly improved our manuscript and we look much forward to your decision.

Sincerely yours,

Bo Brummerstedt Iversen
